# A Comprehensive Study of Piezoelectricity in Chitosan Films

**DOI:** 10.1002/smsc.202500593

**Published:** 2026-03-23

**Authors:** Sofia Papa, Margherita Montorsi, Leonardo Arrighetti, Cristian Rodriguez‐Tinoco, Simone Capaccioli, Laura M. Ferrari, Francesco Greco, Massimiliano Labardi

**Affiliations:** ^1^ The Biorobotics Institute and Department of Excellence in Robotics & AI Scuola Superiore Sant’Anna Pontedera Italy; ^2^ Istituto per i Processi Chimico‐Fisici (CNR‐IPCF) Sede Secondaria di Pisa Consiglio Nazionale delle Ricerche Pisa Italy; ^3^ College of Physics and Optoelectronics Engineering Shenzhen University Shenzhen People's Republic of China; ^4^ Dipartimento di Chimica e Chimica Industriale Università di Pisa Pisa Italy; ^5^ Departamento de Física Facultad de Ciencias Catalan Institute of Nanoscience and Nanotechnology (ICN2), CSIC and BIST Universitat Autònoma de Barcelona Bellaterra Spain; ^6^ Physics Department University of Pisa Pisa Italy; ^7^ Interdisciplinary Center on Sustainability and Climate Scuola Superiore Sant’Anna Pisa Italy

**Keywords:** biopolymers, chitosan, crystals, piezoelectricity, piezoresponse force microscopy

## Abstract

Chitosan films have attracted interest as flexible and biocompatible materials in health monitoring technologies due to their intrinsic piezoelectricity. This work studies, for the first time, the piezoelectric performances of solution‐cast chitosan films in relation to their fabrication process and crystalline content. We investigate how processing parameters, such as solvent type, NaOH treatment duration, and thermal annealing, affect the crystalline content of chitosan films and its correlation with the overall piezoelectric behavior through comprehensive physico‐chemical characterizations. Special care is devoted to a reliable determination of converse piezoelectricity, using constant‐excitation frequency‐modulation piezoresponse force microscopy (CE‐FM‐PFM), a scanning probe method specifically suited to suppress measurement artifacts that affect conventional PFM in the investigation of soft materials such as semicrystalline polymers. Our findings reveal that a post‐processing strategy combining NaOH treatment and thermal annealing significantly enhances the piezoelectric response of chitosan, with a piezoelectric coefficient *d*_33_ reaching 27 pm V^−1^, nearly twice as high as those previously reported in the literature. These results pave a promising path for the development of eco‐friendly and functional piezoelectric materials for biomedical applications.

## Graphical abstract

This work explores the correlation between crystallinity and piezoelectricity in solution‐cast chitosan films with respect to their fabrication process, giving a final recipe allowing to reach a *d*_33_ = 27 pm V^−1^, nearly twice as high as those previously reported in the literature. We investigate how solvent type, NaOH treatment, and thermal annealing influence the crystallinity and piezoelectricity, using constant‐excitation frequency‐modulation piezoresponse force microscopy (CE‐FM‐PFM) to ensure reliable measurements. This study reports a comprehensive physico‐chemical characterization of solution‐cast chitosan films, paving the way for a sustainable route to bioderived and biocompatible piezoelectric materials.

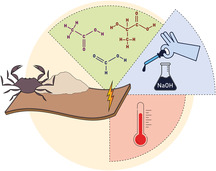

## 1 Introduction

Chitosan is a bio‐derived polysaccharide obtained from the deacetylation of chitin, which is the second most abundant biopolymer after cellulose, extracted from marine crustaceans, insect cuticles, and microorganism cell walls. It consists of randomly distributed β‐(1→4)‐linked glucosamine and *N*‐acetyl‐glucosamine saccharide repeating units, with a low acetyl content. Chitosan has captured significant attention as a low‐cost, bio‐derived, and biodegradable polymer [1] with a considerable potential in biomedical engineering [2, 3], pharmaceuticals [4] and environmental science [5]. Recently, chitosan has been getting attention for its piezoelectric properties [6, 7, 8, 9, 10, 11], which, combined with its low Young's modulus (*E* < 2 GPa), makes it a promising material in the biomedical field of wearable health monitoring. However, chitosan's piezoelectric coefficient is relatively low (*d*_33_ ~ 5 pm V^−1^) compared with the best piezoelectric ceramic materials, e.g. PZT (PbZr_*x*_Ti_1‐x_O_3_, *d*_33_ ~ 500 pm V^−1^), or with the one of poly(vinylidene fluoride) (PVDF, *d*_33_ ~20–30 pm V^−1^), a widely used piezoelectric polymer employed in sensors and flexible devices [12, 13]. Such a low piezoelectric response restricts the advancement of chitosan‐based piezoelectric devices.

Recent studies have proposed various strategies to enhance the piezoelectric behavior of chitosan‐based films. Nicoletti et al. [9] achieved a significant increase in piezoelectricity, up to 18 pm V^−1^, by incorporating chitin nanocrystals into natural chitosan thin films. Alternatively, De Marzo et al. [7] introduced an innovative method to enhance the piezoelectric response without the addition of external materials, relying instead on the prolonged chemical treatment of the chitosan films with NaOH. This methodology also increases their lifespan before biodegradation [8], a key advantage for biomedical applications that require materials to remain stable over extended periods in physiological conditions. An increase in the duration of the NaOH treatment up to 60 min was found to enhance the piezoelectric response *d*_33_ from 5 to 15 pm V^−1^. Further NaOH treatment led to a decrease of piezoelectric response, back to 9 pm V^−1^. This effect could be attributed to modifications in chitosan's crystal structure.

**FIGURE 1:**
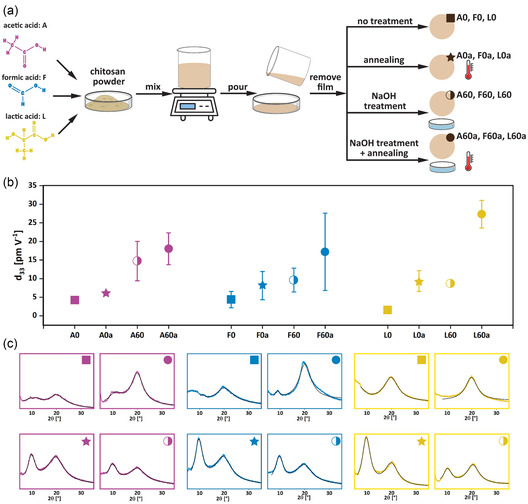
Schematic overview of the experimental workflow. (a) Summary of the fabrication steps of chitosan films, including post‐processing techniques such as NaOH treatment, thermal annealing, or a combination of both. (b) Comparison plot of the *d*_33_ piezoelectric coefficients of chitosan films prepared using three different acids. The letter *a* indicates annealed samples, referring to the temperature at which each acid‐specific sample exhibited the highest *d*_33_ value (200°C for acetic acid (A), 140°C for formic (F) and lactic (L) acids). For each acid, the following conditions are shown: untreated film (A0, F0, L0), annealed film (A0a, F0a, L0a), NaOH‐treated film for 60 min (A60, F60, L60), and films treated with both NaOH and annealing (A60a, F60a, L60a). (c) XRD spectra of the same samples shown in (b), corresponding to the reported *d*_33_ values.

To elucidate how fabrication and post‐treatment parameters influence the piezoelectric behavior of chitosan solution‐cast films and how this relates to their crystalline structure and polymorphism, we performed a comprehensive study schematically summarized in Figure 1.

**FIGURE 2:**
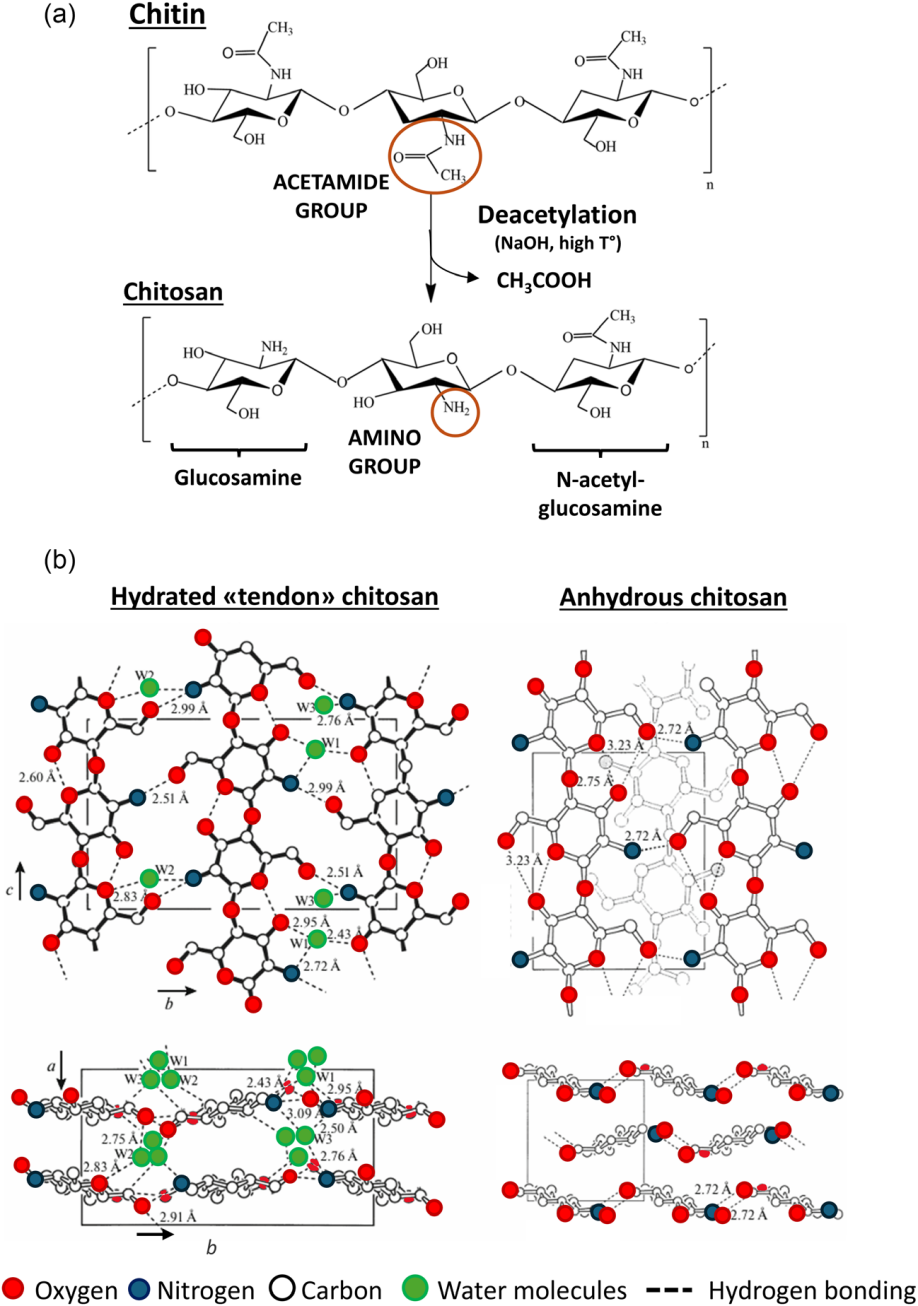
(a) Chemical structure of chitin and chitosan. In the deacetylation process, the *N*‐acetylglucosamines are converted into glucosamine units, having the primary amino group (‐NH_2_) instead of the secondary acetamides (‐NHCOCH_3_). The deacetylation reaction occurs with NaOH at high temperatures. (b) Structure of hydrated and anhydrous chitosan: projections of the layers (top) in a direction parallel to the crystallographic *cb*‐plane and (bottom) along the *a*‐axis, perpendicular to the layers. Reproduced from [14].

To better interpret these relationships, it is helpful to recall the main polymorphic forms of chitosan.Chitosan is a semicrystalline polymer presenting four primary polymorphic forms, namely: (1) hydrated “tendon” form, (2) L‐2 form, (3) “1‐2” form, (4) anhydrous “annealed” form. In polymorphs (1) and (4) (Figure 2), chitosan exhibits an extended twofold helical structure similar to that of chitin or standard cellulose [14]. However, the (1) and (4) polymorphs differ substantially in water content, the only fully anhydrous being the (4), and in their packing density. Chitosan, derived from chitin sources, predominantly adopts the ‘tendon’ polymorphic form (1) [14, 15], which is the most commonly observed one. In this structure, water molecules are embedded within the crystal and stabilize it (Figure 2b). The (2) and (3) polymorphs are obtained as intermediate structures in the irreversible transition from the hydrated crystal form (1) to the anhydrous one (4). This transition may occur under chemical treatment or thermal annealing [14, 15]. Other parameters may influence the crystal structure of chitosan films, such as the acid used as a solvent for its solubilization [16, 17]. Indeed, the structure or size of acids can impact both intramolecular and intermolecular interactions [17].

Since the piezoelectric properties of materials are commonly linked to their crystalline structure, it is essential to investigate the correlation between crystal polymorphism and piezoelectric behavior. In the case of chitosan, a deep understanding of its piezoelectric behavior and how it correlates to the crystalline polymorphism is still lacking.

To address these gaps, we first investigate how various processing parameters, including the type of acid used to prepare chitosan solutions, the NaOH treatment and its duration, and the annealing temperature, affect the crystalline structure of chitosan, as schematically illustrated in Figure 1a. Subsequently, as summarized in Figure 1b, we explore the correlation between the crystalline structure and the piezoelectric response of chitosan films by applying an innovative PFM technique named constant‐excitation frequency‐modulation (CE‐FM) PFM which suppresses electrostatic artifacts [18].

This advanced method overcomes the limitations of traditional PFM, thereby enabling a more reliable assessment of the piezoelectric properties of chitosan. Indeed, most determinations of piezoelectricity conducted on chitosan to date have been performed using PFM operating in contact modality [19]. However, this scanning probe technique is well known to be susceptible to measurement artifacts, especially electrostatic ones, when applied to soft materials, such as amorphous or semicrystalline polymers. As a result, available values obtained for the piezoelectric coefficient of chitosan films should be carefully verified before translating the technology into biomedical applications.

This work is divided into two main parts. The first part focuses on the fabrication of the chitosan films, which were prepared by varying parameters such as the solvent type (acetic acid, formic acid, or lactic acid), the duration of NaOH treatment (0, 30, 60, 90 min), and the annealing temperature (100°C, 140°C, 200°C, 250°C). The impact of these variables and their combination on films’ molecular structure is evaluated by physico‐chemical analysis, using Fourier‐transform infrared spectroscopy in the attenuated total reflection mode (FTIR‐ATR) and thermogravimetric analysis (TGA), as well as morphologically, through atomic force microscopy (AFM) surface analysis. In the second part, we investigate the piezoelectric response and the crystalline structure associated with each fabrication process. The converse piezoelectric coefficients (*d*_33_) of all chitosan films were determined using the CE‐FM‐PFM method [18]. Finally, small‐angle X‐Ray Diffraction (XRD) analysis was performed to correlate our results with the crystal structure of our chitosan films.

This approach aims to establish a clear link between the fabrication and post‐processing parameters of chitosan films, their resulting piezoelectric response, and the associated crystalline structure. Elucidating these relationships is essential for optimizing a fabrication protocol able to significantly enhance the piezoelectric performance of chitosan.

## 2 Results and Discussion

Throughout the text, samples are named with the following convention: the first letter of the acid used for film preparation (A = acetic, F = formic, L = lactic), followed by the duration in minutes of the NaOH treatment (e.g., 0 or 60), and, when applicable, the annealing temperature (e.g., ‘_140’ for samples annealed at temperature *T* = 140°C). For example, ‘A0_140’ refers to a sample prepared with acetic acid, not treated with NaOH, and annealed at 140°C.

### 2.1 Film Fabrication Process and Mechanical Characterization

Twelve film types were realized by varying the employed acid solvent (i.e., acetic acid ‐ A, formic acid ‐ F, and lactic acid ‐ L), and the NaOH bath time (*t* = 0, 30, 60, and 90 min). The NaOH treatment facilitates the removal of residual solvents and deprotonates the NH_3_^+^ groups formed during the interaction between chitosan and the acid used for its dissolution. This process is known to reduce repulsion between polymer chains and to promote the formation of interchain hydrogen bonds, thereby compacting the molecular structure of chitosan. As a result, the mechanical properties of chitosan films are improved, and its solubility is reduced [20]. Figure 1a presents a schematic illustration of the main fabrication steps, while corresponding photographs of such steps are provided in Figure S1.

After the NaOH treatment, the thickness of all films was approximately 30 µm, doubling the original thickness measured before treatment. A macroscopic difference between the untreated films produced with lactic acid (L) and those obtained with acetic (A) and formic (F) acid is evident. Specifically, the L0 films had a more rubbery consistency, likely due to lactic acid's water retention capacity, which is higher compared to that of acetic and formic acids. However, these differences are no longer evident after NaOH treatment, as the process suppresses the residual acid content.

These macroscopic observations are further supported by mechanical analysis. We focused on untreated samples (*t* = 0 min) and NaOH‐treated samples (*t* = 60 min), as the more representative cases. Stress–strain curves were acquired for these samples, and the Young's modulus values are reported in Table 1, while relative stress–strain curves are reported in Figure S2. The obtained data confirm that the chemical treatment significantly increases sample stiffness. The modulus of A0 increases from 3.7 to 5.3 GPa in A60, F0 increases from 2.8 to 5.4 GPa in F60, and L0, which initially exhibits a very low modulus of 1 MPa, increases to 6.4 GPa in L60.

**TABLE 1:** Young's modulus of chitosan films before (A0, F0, L0) and after (A60, F60, L60) NaOH treatment.

Sample	Young modulus, GPa
A0	3.7
A60	5.3
F0	2.8
F60	5.4
L0	0.001
L60	6.4

Notably, the extremely low modulus of L0 highlights the effect of a larger water content in the lactic‐acid‐based films, which softens the material and increases its flexibility. After NaOH treatment, this effect is suppressed, and the mechanical properties of L60 become comparable to those of A60 and F60, indicating that water and residual acid strongly influence the initial mechanical behavior.

The chitosan films were annealed at *T* = 100°C, 140°C, 200°C, and 250°C to investigate the effect of temperature on their structural and physical properties. Each sample was annealed in vacuum for 1 h at the designated temperature. Figure S3 shows the appearance of all the chitosan films after annealing at different temperatures, where an evident color change was observed in all samples. As the temperature was increased, the films progressively darkened, probably due to a Maillard reaction [21].

### 2.2 Morphological Characterization

**FIGURE 3:**
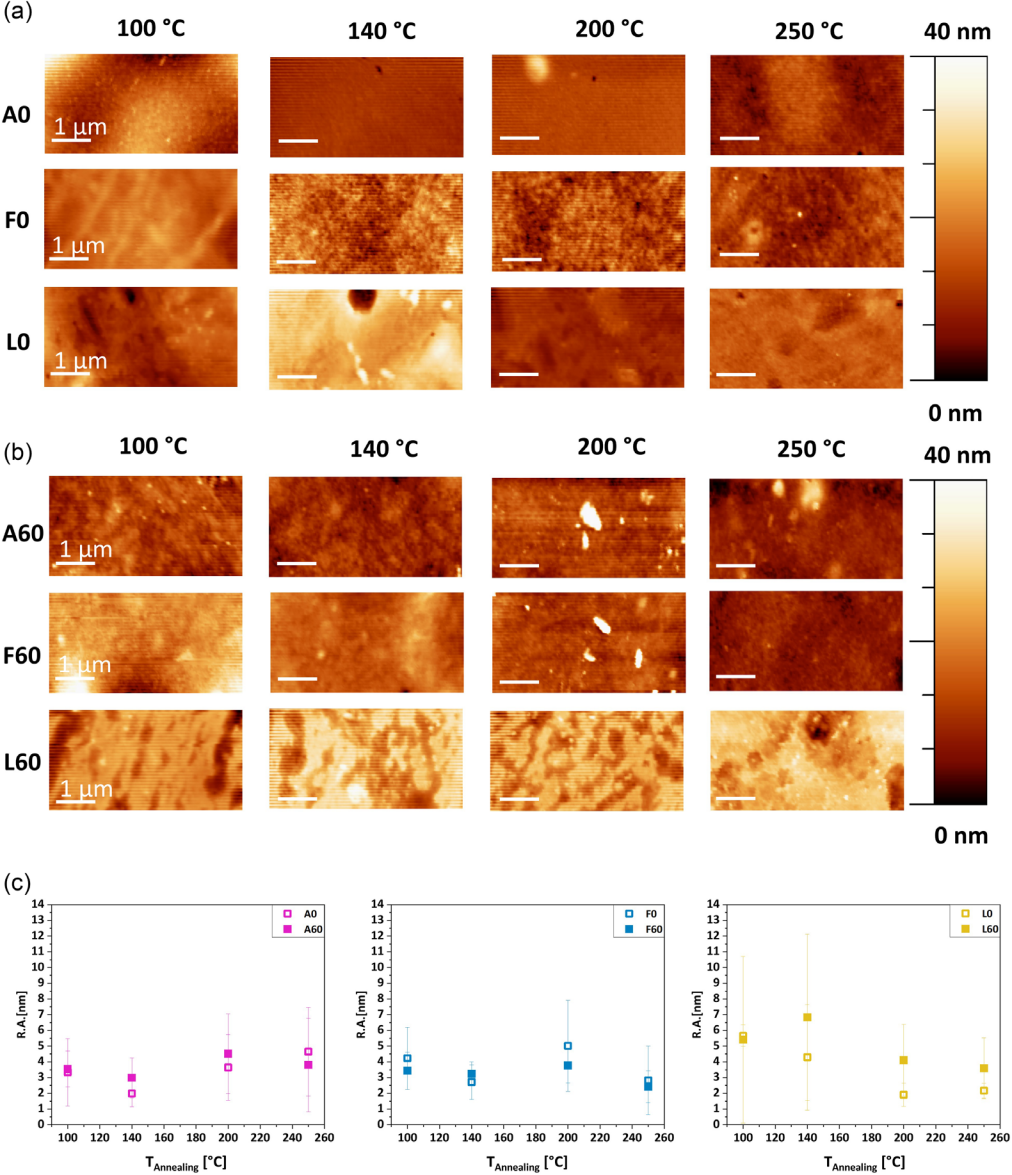
Representative AFM topographic images of chitosan films annealed at different temperatures: 100°C, 140°C, 200°C, 250°C. The annealing temperatures (in °C) are indicated at the top of the figure. (a) Non‐NaOH‐treated films (A0, F0, and L0). (b) NaOH‐treated films (A60, F60, and L60). (c) Average roughness (RA) on chitosan films both non‐NaOH treated (A0, F0, and L0) and NaOH‐treated (A60, F60, and L60) as a function of the annealing temperature.

Atomic Force Microscopy (AFM) analysis revealed no significant differences in surface average roughness (RA) before and after NaOH treatment. As shown in Table 2 and Figure 3, the film morphology is not significantly altered by the chemical treatment. Also, the annealing temperature does not seem to affect roughness with a definite trend. An exception is observed for the L samples, where both the roughness and its standard deviation decrease upon annealing. For instance, in the L0_100 sample, the roughness is 5.7 ± 0.7 nm, indicating a more uniform surface than that of the L60_140 sample, where it reaches 5.4 ± 5.3 nm and reveals a higher degree of morphological inhomogeneity. This may be attributed to several factors, such as differences in the arrangement of crystalline domains or a non‐uniform distribution of crystalline and amorphous regions, at least at the surface of the films. With increasing annealing temperature, however, these values tend to decrease, e.g. to 1.0 ± 0.7 nm for L0_200, and 4.1 ± 2.3 nm for L60_200.

**TABLE 2:** Roughness average (RA) value determined by AFM for each chitosan film sample.

Sample	RA, nm	Sample	RA, nm	Sample	RA, nm
**A0_100**	3.3 ± 2.1	**F0_100**	4.2 ± 2.0	**L0_100**	5.7 ± 0.7
**A0_140**	2.0 ± 0.8	**F0_140**	2.7 ± 1.1	**L0_140**	4.3 ± 3.4
**A0_200**	3.6 ± 2.1	**F0_200**	5.0 ± 2.9	**L0_200**	1.0 ± 0.7
**A0_250**	4.7 ± 2.8	**F0_250**	2.8 ± 2.2	**L0_250**	2.2 ± 0.5
**A60_100**	3.5 ± 1.1	**F60_100**	3.4 ± 1.2	**L60_100**	5.4 ± 5.3
**A60_140**	3.0 ± 1.3	**F60_140**	3.2 ± 0.8	**L60_140**	6.8 ± 5.3
**A60_200**	4.5 ± 2.5	**F60_200**	3.8 ± 1.1	**L60_200**	4.1 ± 2.3
**A60_250**	3.8 ± 3.0	**F60_250**	2.4 ± 1.0	**L60_250**	3.6 ± 1.9

### 2.3 Physico‐Chemical Characterization

**FIGURE 4:**
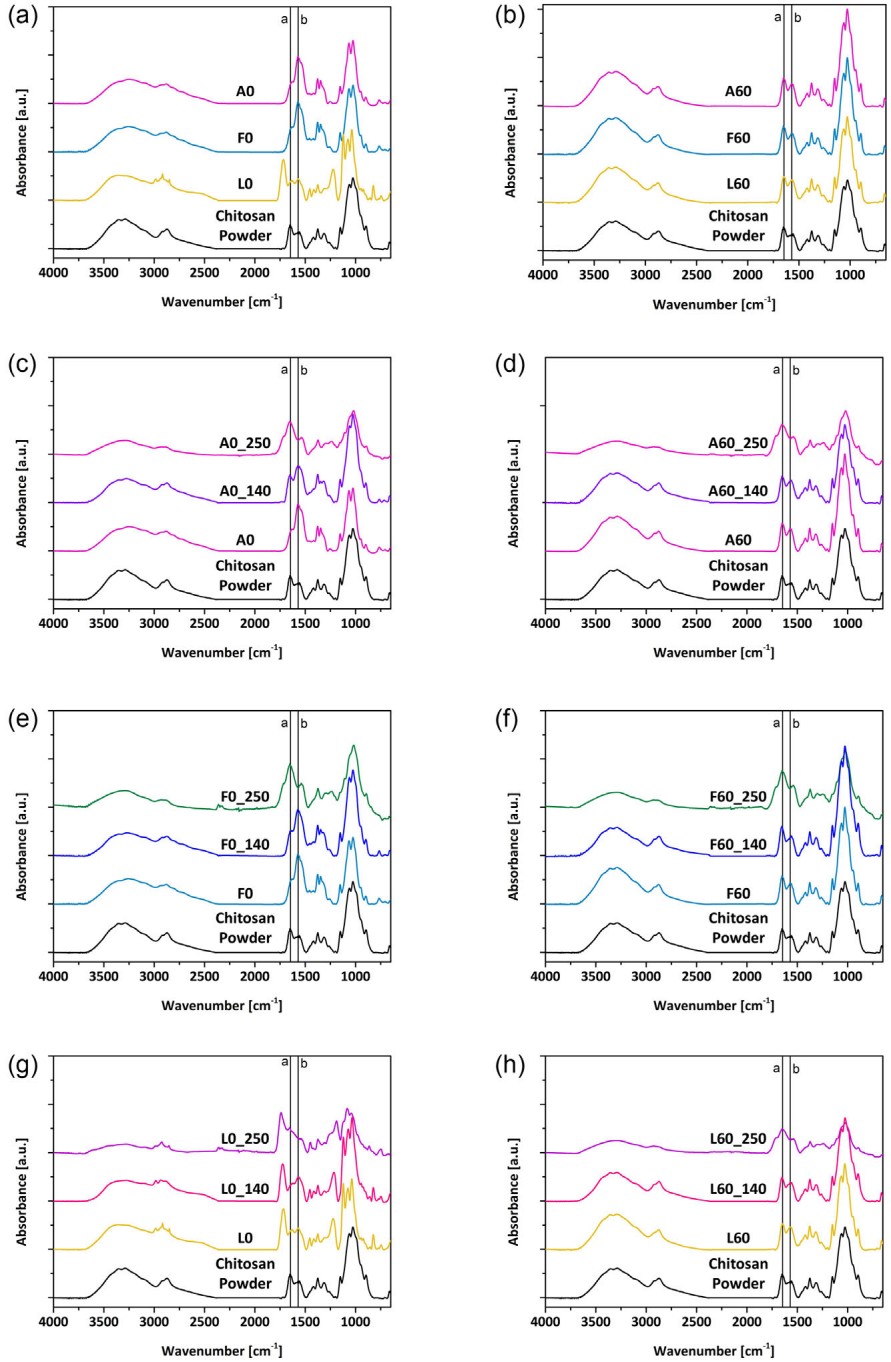
FTIR‐ATR absorption spectra of chitosan films obtained from different film processing conditions (various acids, NaOH treatment, thermal annealing). A spectrum of the chitosan powder is reported as a control. Sample types and annealing conditions are reported in figure legends with the usual nomenclature. “a” and “b” indicate bands at 1645 cm^−1^ and 1570 cm^−1^, respectively.

FTIR‐ATR spectra were acquired to investigate the structural modifications induced in the chitosan films by different processing conditions (type of acid, NaOH treatment, and thermal annealing). The annealing temperatures considered in this analysis were 140°C and 250°C. The acquired spectra are reported in Figure 4. The spectrum of the chitosan powder used to produce our films is also reported as a reference.

FTIR spectra show the characteristic vibrational modes of not fully deacetylated chitosan chains. Specifically, the C=O stretching (amide I) band appears at around 1700–1600 cm^−1^, which is attributed to the NHCO amide groups of residual *N*‐acetylglucosamine. Additionally, the 1600–1500 cm^−1^ band corresponds to the NH_2_ bending of the amine group (amine I), which overlaps with the NH bending band of the amide II [22]. In Figure 4, the centers of the two bands at 1645 (NH_2_ bending) and 1570 (NH_3_^+^ bending) cm^−1^ are indicated respectively with ‘a’ and ‘b’. The broad absorption band centered at 3370 cm^−1^ confirms the overlapping of OH and NH stretching vibrations. The 2923 and 2877 cm^−1^ absorption bands lie in a complex spectral region where several bands have been observed due to symmetric and asymmetric stretching of CH bonds from the saccharide ring, and –CH_2_OH and –CH_3_ groups [23].

Figure 4a displays the FTIR spectra for samples A0, F0, L0, and chitosan powder as a reference. In the L0 spectrum, the overlapping bands of the lactic acid used can be observed. Notably, the presence of carboxylic acid is indicated by distinct features in the stretching regions of the OH and CH bonds, and by the carboxylic group band located at around 1715 cm^−1^. In contrast, the A0 and F0 samples show a significant increase in the band associated with NH_2_ bending, resulting from the amine group's protonation by the acid. When protonated in its NH_3_^+^ form, the bending or ‘scissoring’ deformation of the amine group is more evident, since it is no longer involved in interchain bonding, because of electrostatic repulsion. This results in an increased band intensity at 1570 cm^−1^. The spectra of F60, A60, and L60 (Figure 4b) fully recover the original form of the chitosan powder reference spectrum. This finding confirms that the NaOH treatment effectively removes the acid in all three cases studied (A, F, L), completely recovering the initial deprotonated form [24, 25].

In all cases, the broad band of the OH and NH stretching increases in intensity after the NaOH treatment, due to the presence of absorbed and hydrogen‐bonded water molecules and increased interchain hydrogen‐bonding interactions, which are increased by removing the acid steric footprint and by amine deprotonation.

Figure 4a,c,e,g display spectra of the non‐NaOH‐treated samples. Annealing at 140°C does not influence the chemical structure of the film, with the peak of amine's protonation at 1570 cm^−1^ just slightly changing with respect to reference chitosan, depending on the specific acid used for chitosan solubilization (Figure 4c,e,g). On the contrary, after annealing at 250°C, a strong absorption band at 1645 cm^−1^ with a shoulder at 1717 cm^−1^ appears, indicating the formation of the new carbonyl moieties due to oxidation [23]. This is also confirmed by the darkening of films, as visible from the pictures of all samples shown in Figure S3. The same trend is observed in NaOH‐treated samples (Figure 4b,d,f,h). Of note, the broad band of OH stretching is not affected by the annealing at 140°C, highlighting the stable and strong bonding between chitosan chains and the interchain water. During the annealing at 250°C, the intensity of this band slightly decreases, indicating an initial stage of dehydration. NH_2_/NH_3_^+^ ratios, calculated from the intensities of the vibrational modes, ‘b’ and ‘a’ respectively, are reported in Table S1.

Overall, these results suggest that the NaOH treatment is crucial for removing acidic residuals, through a neutralization process, and thereby reducing repulsion between polymer chains. It promotes the formation of interchain hydrogen bonds, which compact the molecular structure of chitosan, reducing the film's solubility [20].

Focusing on the NH_2_/NH_3_^+^ ratio provides valuable information about the molecular structure of the chitosan films, which in turn may affect their piezoresponse. When the NH_3_^+^ band is prominent, the amine group is less involved in interchain bonding due to electrostatic repulsion. This suggests that, in the protonated state, since interchain interactions are hindered, a more disordered arrangement of polymer chains occurs, as well as a more random alignment of dipoles. Conversely, an increase in the ratio discussed above is associated with increased interchain hydrogen‐bonding interactions, which are expected to promote a more compact film structure and possibly a better alignment of dipoles, favoring piezoelectricity.

**FIGURE 5:**
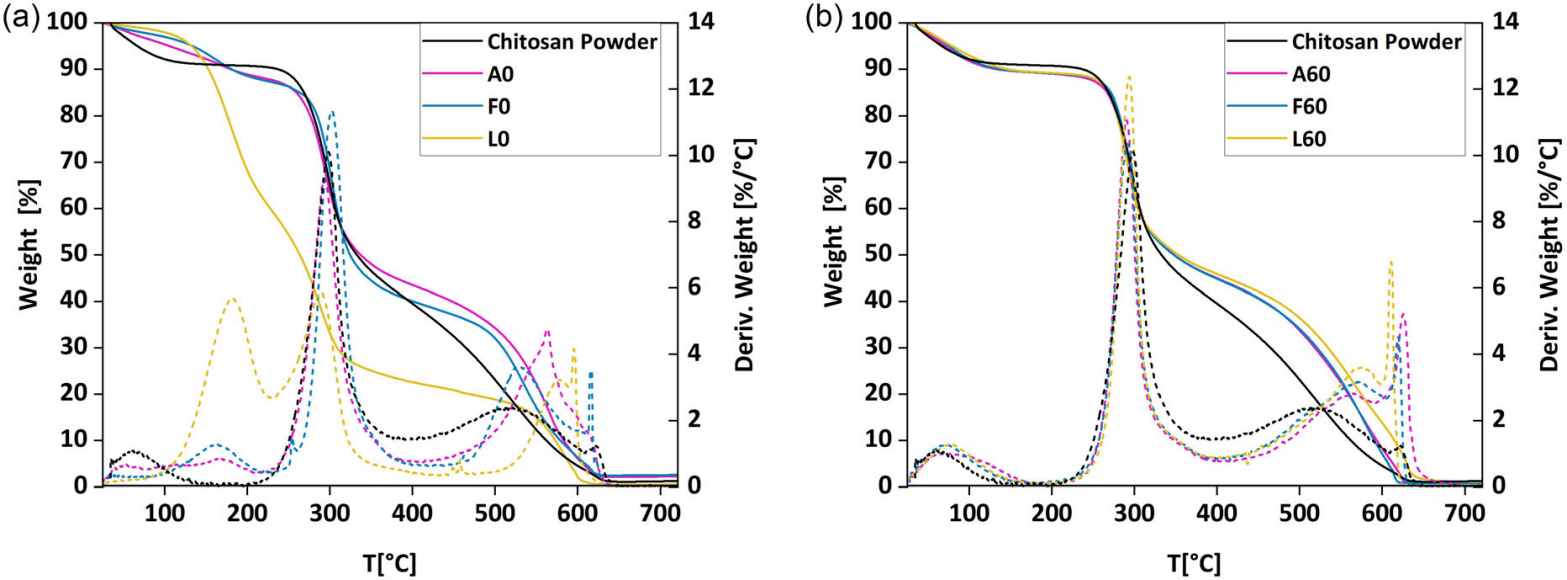
TGA thermograms of chitosan films obtained in air atmosphere at a heating rate of 10°C/min. (a) Weight loss profile (%, solid lines) and its first derivative (%/°C, dashed lines) of control films from solution casting of chitosan in acetic (A0), formic (F0), and lactic (L0) acids, and chitosan powder for comparison. (b) Weight loss profile (%, solid lines) and its first derivative (%/°C, dashed lines) of NaOH‐treated films, and chitosan powder for comparison.

In Figure 5, the thermogravimetric analysis of chitosan films is reported for non‐treated (a) and NaOH‐treated (b) samples. In all the thermograms, we can identify three main temperature ranges, namely I from 25°C to 220°C, II from 220°C to 400°C, and III from 400°C to 650°C. The weight loss observed in all samples in range I is due to the evaporation of physically absorbed water and residual acid molecules from the films. A and F samples exhibit a weight loss of 12% in this range, a value close to that of the pristine chitosan powder (9%) and consistent with the water content of films exposed to ambient air [26]. Differently, L samples show a significantly higher weight loss in this range, reaching 40%, suggesting a greater tendency of lactic acid to bind to the chitosan chains and retain water molecules compared to the other acids. This hypothesis is further supported by thermograms of NaOH‐treated samples (Figure 5b). In this case, as expected due to the successful neutralization, the three samples behave more similarly, with weight losses of 10%, 10%, and 11% for A60, F60, and L60, respectively.

The weight loss in range II can be attributed to the degradation of the saccharide structure of chitosan. This includes dehydration of saccharide rings, depolymerization, and decomposition of acetylated and deacetylated chitin units. In this region, the weight loss of A0, F0, and L0 samples is 49%, 45% and 38%, respectively, while for A60, F60 and L60 ones is 44%, 43%, and 43%, respectively. The weight loss in range III is observed only upon TGA in air, indicating that such mass loss is associated with the oxidative degradation of the carbonaceous residue formed during the earlier decomposition stages. Although we could not provide a clear explanation for this behavior, the data in range III indicate that chitosan films are more temperature‐resistant than chitosan powder, possibly due to their more compact initial structure, especially after NaOH treatment. All the results of thermogravimetric analysis are summarized in Table S2.

### 2.4 Crystallization and Piezoresponse: Correlation Study

#### 2.4.1 PFM

The converse piezoelectric coefficients, *d*_33_, provide the strain *s*_3_ of an unbound piezoelectric material when subjected to an electric field *E*_3_ along the same direction. The *d*_33_ coefficients of our chitosan films were determined by PFM. Here, an electric potential is applied to the conductive probe tip of an atomic force microscope (AFM), and the surface displacement taking place in the sample, due to its thickness change under the applied electric field and the generated stress, is detected by the AFM force probe [19]. In this work, we applied a peculiar PFM configuration, named constant‐excitation frequency‐modulation (CE‐FM‐PFM) [18], devised for application to compliant materials like amorphous or semicrystalline polymers. Indeed, it is well established that PFM results can be severely affected by measurement artifacts [18]. This issue must be carefully considered, especially in soft samples, where electrostatic forces, due to the tip polarization, can induce deformations that can be erroneously assigned to the effect of piezoelectricity. Reducing such artifacts is particularly crucial when dealing with materials exhibiting low piezoelectric coefficients, i.e., of the order of a few pm V^−1^.

The CE‐FM‐PFM method inherently reduces electrostatic background in piezoresponse detection by using non‐contact mode AFM cantilevers with spring constants of several tens of N m^−1^, thereby reducing cantilever bending due to electrostatic forces, that mimics the effect of piezoelectric deformation. This contrasts with standard contact‐mode PFM, where spring constants are typically only a fraction of N m^−1^ to prevent damage to soft surfaces and enable contact‐resonance methods [27] for enhancing low piezoresponse signals. Nevertheless, a residual electrostatic background of a few pm V^−1^ could persist. Therefore, further artifact subtraction methods should be applied especially when dealing with small piezoelectric yields. For thick polymer films, a background estimation can be obtained by slightly withdrawing the probe from the surface, to bring it out of the repulsive AFM interaction regime, thereby suppressing the effect of piezoresponse while the electrostatic contribution is still retained [18]. The quantitative piezoresponse yields reported in the literature for several materials are exclusively determined by PFM [7]. Therefore, misinterpreting such results can lead to inaccurate conclusions. In this work, particular care has been devoted to the correct determination of piezoresponse by PFM.

**FIGURE 6:**
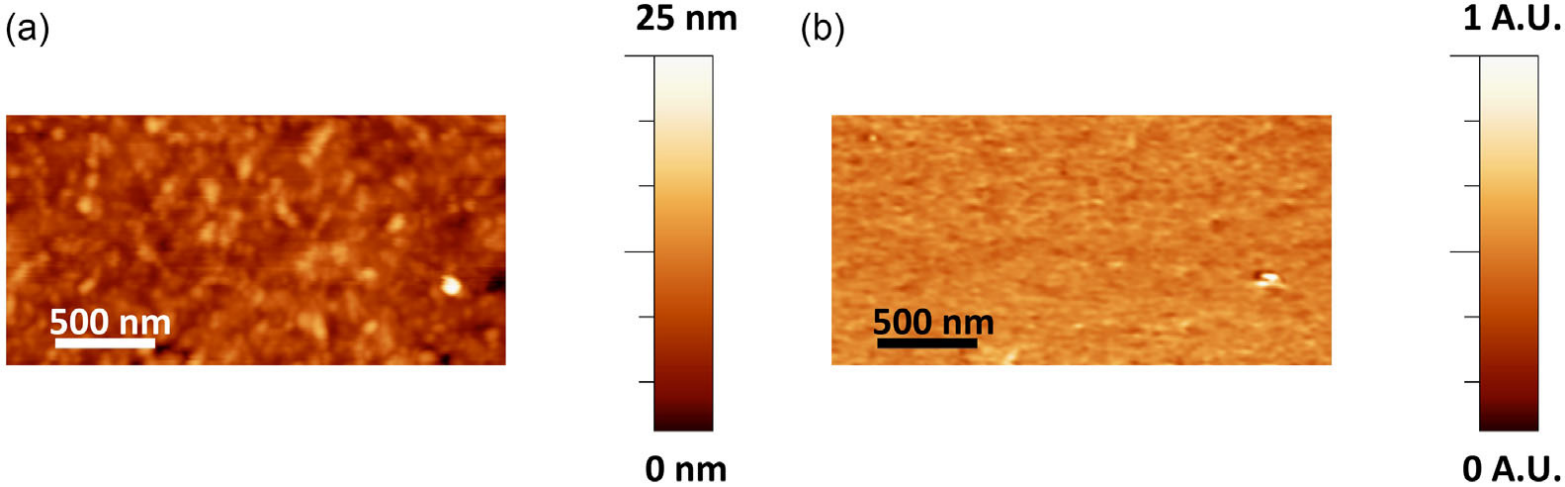
Representative (a) topography and (b) PFM measurement images of sample A60_200.

A typical example of CE‐FM‐PFM measurement images is shown in Figure 6, where it is clearly evident that it is possible to reach nanoscale resolution in a non‐destructive manner.

**FIGURE 7:**
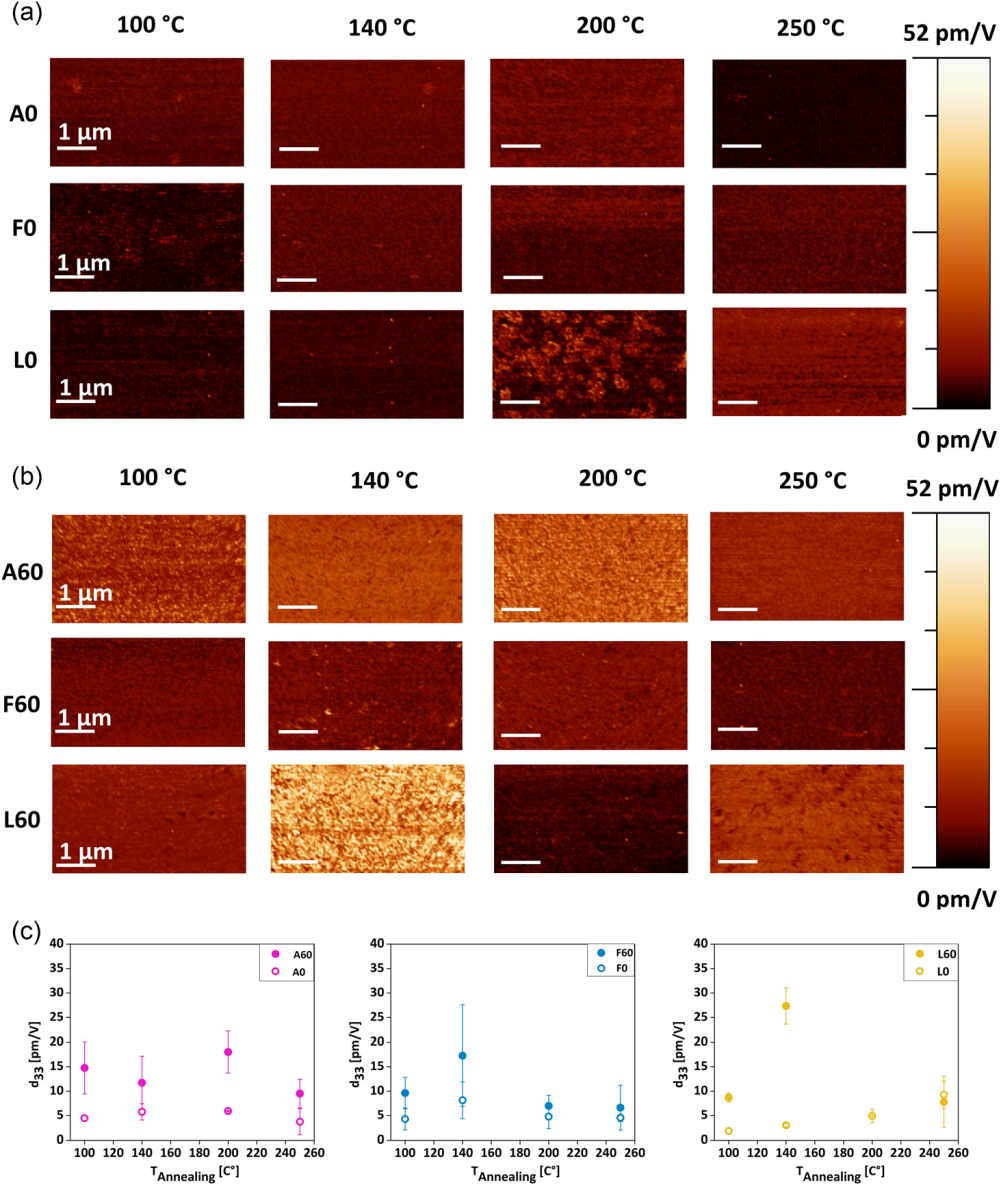
Representative PFM images of chitosan films annealed at: 100°C, 140°C, 200°C and 250°C. The annealing temperatures (in °C) are indicated at the top of the figure. (a) Non‐NaOH treated chitosan films (A0, F0, and L0). (b) NaOH treated chitosan films (A60, F60, and L60). (c) Average *d*_33_ coefficient of chitosan films both non‐NaOH‐treated and NaOH‐treated made using acetic acid (A0, A60), formic acid (F0, F60), and lactic acid (L0, L60), as a function of annealing temperature.

Figure 7 presents the PFM images and the calibrated piezoelectric response (*d*_33_ coefficient) of all the chitosan films analyzed, measured via CE‐FM‐PFM, as a function of the annealing temperature. All PFM images were taken by applying an AC voltage of 5 *V*_rms_ (∼7 *V*_p_), at a frequency of around 160 Hz. In Figure 7a,b CM‐FM‐PFM images acquired over an area of 5 × 2.5 µm^2^ are shown. The maps display a homogeneous piezoelectric response across the entire scanned area for all samples. A darker image is clearly observed for samples A0, F0, and L0 (Figure 7a) compared to the NaOH‐treated samples (Figure 7b), indicating an overall higher *d*_33_ value after the chemical treatment. Among the NaOH‐treated samples, the brightest image corresponds to L60, as this is the sample with the higher *d*_33_ value.

The *d*_33_ values obtained from the statistical analysis of the CM‐FM‐PFM images are reported in the plots of Figure 7c. Measurements were performed as follows: for each chemical‐treated and non‐treated sample, two independent specimens were analyzed (for a total of 12 specimens). PFM measurements were then conducted on each sample type at four different annealing temperatures (for a total of 24 measurements). For each annealing condition, three images, as the ones in Figure 6a,b, were collected at different positions across the sample area. The average *d*_33_ value and standard deviation were first calculated for each individual image; subsequently, the mean value and standard deviation were determined by combining the results obtained from all images for each data set. In A and F chitosan films, treated and untreated samples follow similar temperature‐dependent trends. A0 and A60 samples reach peak *d*_33_ values after annealing at 200°C, while F0 and F60 peak at 140°C. In contrast, L films exhibit distinct behaviors: L0 shows a monotonic increase with temperature, whereas L60 reaches its maximum after annealing at 140°C. As indicated by TGA analysis, this divergence likely arises from the higher water retention of L0, which may affect crystallinity or induce different polymorphs. Among the NaOH‐treated samples, A60_200 (18.0 ± 4.3 pm V^−1^) and F60_140 (17.3 ± 10.4 pm V^−1^) show comparable piezoelectric responses, though the latter appears less uniform over the film area. Indeed, the larger error observed for F60_140 *d*_33_ coefficient may be due to a less homogeneous distribution of crystalline regions across the film. The best piezoelectric performance is observed in L60_140, with a *d*_33_ of 27.3 ± 3.7 pm V^−1^, almost double the typical values reported for chitosan films in the literature [7, 9], and it appears to be relatively uniform across the sample. These findings demonstrate that the combination of NaOH chemical treatment and controlled thermal annealing can significantly enhance the piezoelectric performance of chitosan films, likely by promoting a higher degree of piezoactive crystalline domains by influencing the formation of specific polymorphs.

#### 2.4.2 XRD

To investigate the correlation between the specific fabrication process of the chitosan film, the piezoelectric yield, the crystallinity degree, and the crystal polymorphism, small‐angle X‐Ray Diffraction (XRD) patterns were acquired. By observing the XRD spectra of the chitosan films reported in Figure S4, one can notice that all chitosan samples exhibit peaks at *2θ* = 20° and *2θ* = 10°, the latter being absent only in the L0 sample. These results are consistent with chitosan XRD spectra reported in literature [11, 28, 29]. The two peaks correspond to the (020) and (110) crystallographic planes of the hydrated form of chitosan, which crystallizes in an orthorhombic unit cell (space group P2_1_2_1_2_1_).

All samples, both before and after the NaOH treatment, exhibit XRD peaks at a consistent angular values. We did not observe a clear transition from the hydrated to the anhydrous polymorph, as reported in some literature [28]. Instead, our data suggest that the thermal and chemical treatments primarily affect the formation and ordering of a specific polymorphic phase, namely, the one related to the coexistence of the peaks at 10° and 20°, rather than triggering a clear phase transition between hydrated and anhydrous forms. One possible explanation is that our chitosan films exhibit a more stable polymorphic structure, possibly due to differences in initial molecular organization, residual solvent interactions, or processing conditions. Additionally, we observe that the significant broadening of the XRD peaks obtained, particularly in comparison with those reported in previous studies [28], reflects a substantial amorphous contribution, which may obscure the appearance of new crystalline features. It is also plausible that the low‐order degree of crystals in our films reduces the possibility of a distinct phase separation, making the anhydrous signature less pronounced or undetectable under our experimental conditions.

The peak at *2θ* = 10° is relatively small in samples not subjected to NaOH treatment, but it becomes more pronounced after thermal annealing. This suggests that thermal annealing and NaOH treatment have similar effects on the crystalline structure, albeit to a different extent, confirming the findings of the FTIR analysis. For all NaOH‐treated samples, a noticeable presence of the peak at *2θ* = 10° is observed, with its intensity varying with thermal annealing, although without a clear trend. Since the changes of the XRD pattern indicate variations in the crystalline structure of the chitosan films, we have chosen to consider the ratio between the intensity of the peak at 10° and the one at 20° (*I*_10_ */I*_20_) as an indication of polymorph change. Concerning the total degree of crystallinity of polymers from XRD spectra, although a variety of methods have been developed for its determination [30], we adopt here a simple approach where the degree of order in the crystalline structure is related to the broadening of peaks. This is done by defining an estimator *C*_*d*_ of the degree of crystallinity, as the weighted average of peak sharpness, defined as *Q = θ*_0_/Δ*θ*, with *θ*_0_ the average diffraction angle and Δ*θ* the peak width derived from a Lorentzian fit, as follows:

(1) $$C_{d}=\frac{Q_{1}\times A_{1}+Q_{2}\times A_{2}}{A_{1}+A_{2}}\alpha$$

where *Q*_1_ and *A*_1_ are, respectively, the sharpness and the area under the peak at *2θ* = 10°, *Q*_2_ and *A*_2_ are referred to the peak *2θ* = 20°, and *α* is a normalization factor. This estimation is made under the assumption that the broader the peak, the greater the contribution of the amorphous region to the overall distribution. Indeed, it is observed that XRD spectra of amorphous chitosan samples show very broad peaks, although still centered at the same angles [29].

**FIGURE 8:**
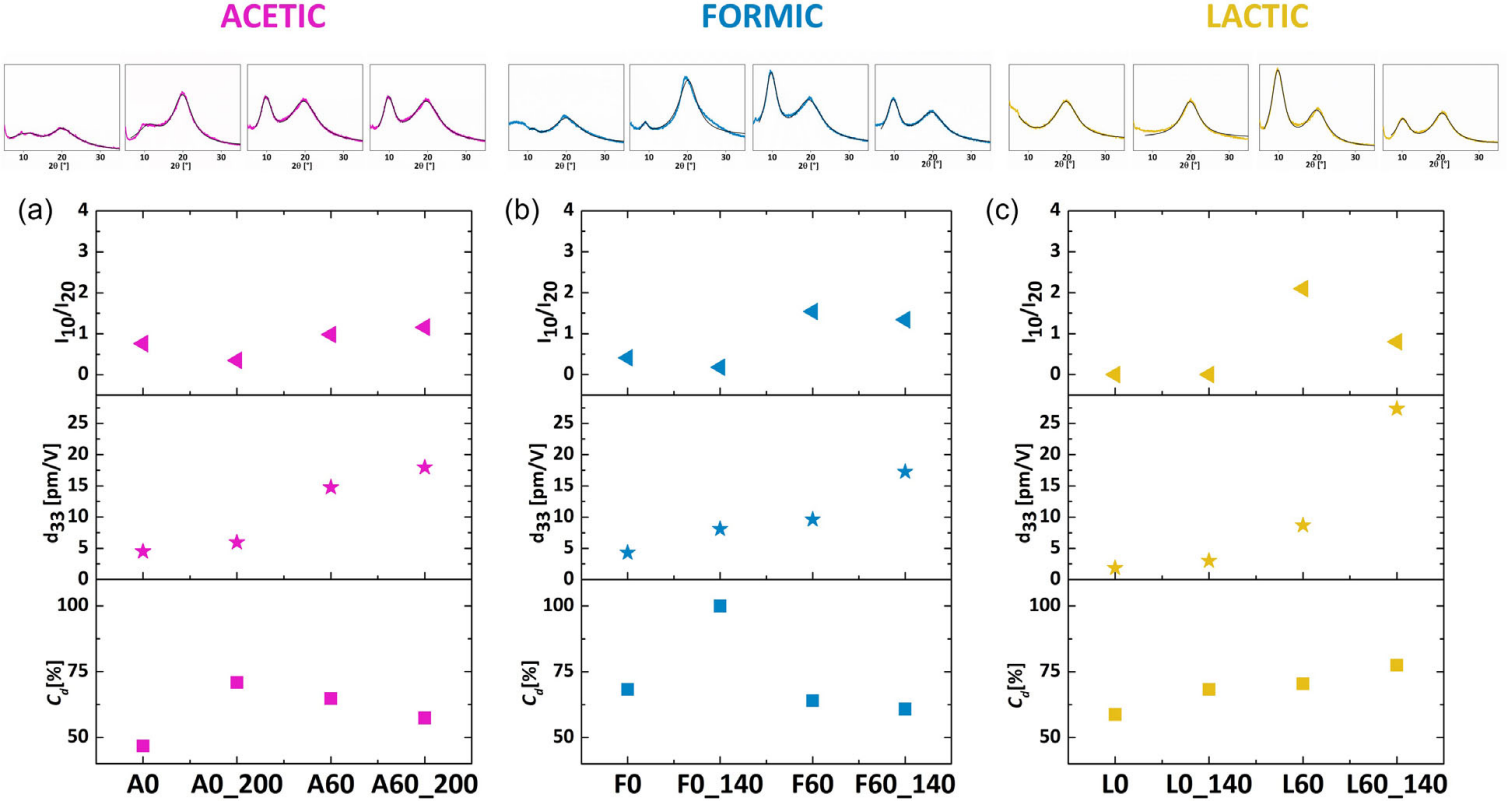
Thumbnails of XRD spectra, ratio of the XRD peaks at 10° and 20° (*I*_10_ */I*_20_), *d*_33_ coefficient, and crystallinity degree *C*_*d*_ for NaOH untreated and treated samples before (A0, F0, L0) and after (A0_200, F0_140, L0_140) annealing, for chitosan from (a) acetic (A), (b) formic (F), and (c) lactic (L) acid.

Figure 8 summarizes the results obtained from XRD on all samples, where spectra are schematically reported as thumbnails, by comparing the *I*_10_ */I*_20_, the *C*_*d*_ value, and the corresponding *d*_33_ values obtained in PFM measurements. The values of the *d*_33_ coefficients, crystallinity degrees, and *I*_10_ */I*_20_ for all samples are provided in Table S4. It is difficult to find a clear correlation between the *d*_33_ values and the degree of crystallinity, *C*_*d*_. For instance, the sample A0_250, which presents the highest *C*_*d*_, exhibits a low piezoelectric response (*d*_33_ = 3.7 ± 2.7 pm V^−1^). The correlation coefficient between crystallinity and piezoelectric response is −0.15, indicating a weak and statistically non‐significant relationship between them. However, it is evident that, for each acid, both the *d*_33_ and the *I*_10_ */I*_20_ values are higher in NaOH‐treated samples, both before and after thermal annealing. Although the trends are not identical across Figure 8a–c, the *d*_33_ values for samples non‐treated by NaOH are the lowest for all the acids used. For this reason, we evaluated the correlation between *I*_10_ */I*_20_ and the *d*_33_ coefficient. In this case, the correlation coefficient was 0.4, with a *p*‐value of 0.052, suggesting a moderate but significant correlation. This result leads us to conclude that, to effectively harness the piezoelectric properties of chitosan, it is crucial to target the formation of a specific crystal polymorph, identified as the ratio between the intensity of the peak at 10° and the one at 20° (*I*_10_ */I*_20_), instead of merely concentrating on the overall crystalline content of the sample. The underlying reason may be that, within a given crystalline structure, dipoles can adopt orientations that are more favorable for piezoelectric activity. This does not imply that crystallinity and polymorphism act in opposition; rather, a certain degree of crystallinity is a prerequisite for the polymorphic arrangement to influence the material's properties. In this framework, crystallinity provides the structural basis, while the specific polymorphic organization may determine how effectively dipoles are aligned and contribute to the *d*_33_ response.

Additionally, we find that the best piezoelectric performance is achieved with a proper combination of NaOH treatment and thermal annealing.

## 3 Conclusions

This work presents a comprehensive study on the piezoelectric behavior of cast chitosan films at variance of solvent type, chemical treatment, and thermal annealing. By systematically tuning such key processing parameters, we gained deeper insight into how chitosan's molecular organization can determine its piezoelectric behavior. These findings provide both a practical route to performance improvement and a clearer understanding of the structure–property relationships that govern chitosan's functionality.

In particular, FTIR analysis revealed that a 60‐minutes NaOH treatment and high‐temperature annealing (*T* ≥ 140°C) have similar effects on chitosan films, primarily by promoting the removal of acids and enhancing interchain hydrogen bonding. However, the chemical treatment proved to be more effective than annealing at *T* ≤ 200°C, whereas annealing at 250°C produced effects comparable to those of the chemical treatment.

No significant differences were observed between films fabricated with different acids, except for those made with lactic acid, which exhibit distinct behavior when non‐NaOH‐treated, due to increased water retention.

Our findings indicate that all the chitosan films, produced with the three different acids, exhibit a remarkable increase in their piezoelectric response after chemical neutralization treatment by NaOH.

The highest *d*_33_ coefficient for each acid is achieved with a combination of chemical treatment and thermal annealing. Specifically, the combination used for the L60 sample resulted in a notable *d*_33_ = 27 pm V^−1^, comparable to that of PVDF [13].

When correlating structural parameters (through XRD measurements) with piezoelectric response (through CE‐FM‐PFM measurements), it becomes evident that the piezoelectric response of chitosan films does not just depend on their overall crystallinity, but rather on the presence (and relative abundance) of different polymorphs. Our findings do not indicate a transition between distinct polymorphic forms, but rather the progressive formation and stabilization of a specific polymorph under defined processing conditions. The processing routes that promote the development and stabilization of a specific crystal polymorph, rather than merely increasing the total degree of crystallinity, are therefore the best way to enhance chitosan films’ piezoelectricity.

In view of developing a functional resonator device, understanding of the mechanical properties of chitosan films becomes crucial. Recent works highlight the elastomeric properties of chitosan films as promising mechanical features for the development of wearable and soft piezoelectric devices [9]. However, a certain degree of rigidity is essential for wearable piezoelectric films to work effectively as resonators [31, 32]. This rigidity is typically associated with the material's crystallinity. In this context, the NaOH treatment plays a key role, as it deprotonates the amino groups of chitosan (converting –NH_3_^+^ to –NH_2_), thereby decreasing the material's hydrophilicity and water uptake, resulting in enhanced structural stability and stiffness as illustrated in Table 1. This not only enables acoustic characterization of chitosan‐based devices in aqueous environment, which is otherwise not feasible with untreated, highly hydrophilic chitosan, but also improves the films’ suitability for wearable devices, as well for biomedical applications. In particular, the reduced hydrophilicity minimizes the plasticizing effect of water, preventing undesired swelling or softening upon contact with the skin's moisture and thereby maintaining the film's mechanical integrity and stable functional piezoelectric performance during use.

To move toward a complete understanding of the device's performance, work is in progress including Dynamic Mechanical Analysis (DMA) and Broadband Dielectric Spectroscopy (BDS) [33], enabling correlation between thermal transitions (*T*_g_), electrical and electromechanical behavior, and mechanical properties.

In conclusion, the present study offers an efficient strategy for obtaining the polymorphic phase of chitosan which exhibits the highest piezoelectric properties reported to date, comparable to those of synthetic and poorly biodegradable piezopolymers. This may represent a significant advancement for developing chitosan‐based materials in the context of eco‐friendly flexible and wearable electronics, where tailored material properties are crucial for device performance.

## 4 Materials and Methods

### 4.1 Fabrication of Chitosan Films

The molecular weight distribution of medium molecular weight (MMW) chitosan (Sigma–Aldrich, Saint Louis, MO, USA) was determined using Gel Permeation Chromatography (GPC). The chromatographic separation was performed on an Agilent PL Aquagel‐OH 40 80 μm column (300 × 7.5 mm). The mobile phase was composed of 0.5 M NaNO_3_ and 0.01 M NaH_2_PO_4_·2H_2_O, with the pH adjusted to 2.33 using orthophosphoric acid. Samples were prepared at a concentration of 0.5% w/v in a 1% v/v acetic acid solution. The system operated at a constant flow rate of 1.0 mL/min with an injection volume of 100 μL. Detection was performed via a UV detector set at 210 nm. Molecular weight values were derived from a calibration curve established using polyethylene oxide (PEO) standards. Based on the calibration curve, the M_w_ (Weight‐average molecular weight) and M_*n*_ (Number‐average molecular weight) of the chitosan sample were 259.39 and 197.87 kDa, respectively, resulting in a PDI (Polydispersity index) of 1.31. This relatively low PDI indicates a high degree of macromolecular homogeneity for a natural polysaccharide.

Solutions were prepared by adding MMW chitosan 1% (w/v) and different acids 2% (v/v) to distilled water. The employed acids were acetic, formic and lactic acid (all from Sigma–Aldrich). The solution was stirred overnight and then centrifuged at 3500 rpm for 1 h to remove impurities. The solution was then poured into circular Petri dishes of 45 mm in diameter, as shown in Figure S1a, using 6 mL of solution per centimeter square of area, calculated to achieve a film thickness of 30 µm after the post‐processes. After 6 h in the oven at 60°C, the films were left to dry overnight at room temperature. Once dried, films were immersed in a 1 M NaOH solution bath for neutralization, for different amounts of time (30, 60, and 90 min). Non‐neutralized films were used as controls. After this step, the obtained films (Figure S1b) were rinsed with distilled water, and their pH was measured using pH indicator strips until it reached 7. Once the films were washed, they were placed on a flat surface (i.e., the bottom of a Petri dish, as shown in Figure S1c) to dry as flat, thin films, at room temperature for 3 h. All the analyzed films were pre‐annealed for 1 h at 100°C under vacuum to ensure a fair removal of residual water and solvents.

### 4.2 Thickness Gauge

The thickness of each free‐standing film was measured using a digital caliper (543 Digital ABSOLUTE Indicator, Mitutoyo), averaging the measurements from 10 different film locations.

### 4.3 Mechanical Testing

An Instron 5965 series universal testing machine equipped with a 10 N load cell was employed for mechanical properties assessment. The testing protocol included a single block of increasing strain. The elongation at break values were evaluated as the maximum elongation reached before breaking. The Young's modulus *E* was determined from the stress–strain curves by analyzing the linear portion corresponding to the elastic region of the material.

### 4.4 FTIR

The infrared spectra were collected with an FTIR Nicolet IS20 spectrometer (Thermo Scientific, Waltham, MA, USA) in attenuated total reflection (ATR) mode. Each spectrum was obtained in the wavenumber range 4000–650 cm^−1^ by averaging 16 scans at a resolution of 4 cm^−1^. The Omnic Specta (software Thermo Fisher Scientific, Madison, WI, USA) was used to collect the data. Origin Pro 9 software was used for data processing and visualization. The results are shown as the average of three spectra per sample after baseline correction. The band at 2868 cm^−1^ was used as a normalization reference [34].

### 4.5 TGA

TGA analysis was performed by a SII TG/DTA 7200 EXSTAR Seiko analyzer (Seiko, Chiba, Japan) under air atmosphere, with a flow rate of 200 mL/min for all measurements. The analysis was conducted on 5–10 mg of sample over the temperature range from 30°C to 700°C at a 10°C/min rate in an alumina pan.

### 4.6 AFM and PFM

Both AFM and piezoresponse force microscopy in CE‐FM mode were performed by a Multimode atomic force microscope with a Nanoscope IIIa controller (Veeco Instruments Inc., Sunnyvale, CA, USA) equipped with an ADC5 extension, TAC temperature controller, and a gas cell for environment control. Unless specified, all measurements were performed in a dry N_2_ atmosphere and at 37.00°C ± 0.03°C. Frequency modulation mode was implemented by adding a PLLPro2 AFM controller (RHK Technology Inc., Troy, MI, USA), an SR830DSP digital dual‐phase lock‐in amplifier (Stanford Research Systems, Sunnyvale, CA, USA), and additional home‐built electronics for signal redirection. Used cantilevers were from NanoSensors (Neuchatel, Switzerland; Model PPP‐NCLPt, platinum/iridium coated silicon, resonant frequency around 150 kHz, spring constant around 40 N/m). The roughness analysis reported in Table 2 was performed by means of the AFM as follows: for each chemical‐treated and non‐treated sample, two independent specimens were analyzed (for a total of 12 specimens). AFM topographical characterization was conducted on each sample type at four different annealing temperatures (for a total of 24 measurements). For each annealing condition, three images, as the ones reported in Figure 3a,b, were collected at different positions across the sample area. The average roughness value and standard deviation were first calculated for each individual image; subsequently, the mean value and standard deviation were determined by combining the results obtained from all images for each data set.

All our PFM images were taken by applying an AC voltage of 5 *V*_rms_ (∼7 *V*_p_), at a frequency of around 160 Hz*.*

Further details of the measurement principle and setup can be found in the Supporting Information and in Reference [18].

The films were annealed in situ using the built‐in AFM thermal controller for 1 h, under a dry N_2_ atmosphere unless specified. The AFM probe holder was removed and replaced by an empty one during annealing to prevent probe contamination from sample due to degassing. Successive PFM images were taken at constant temperature over a 5 × 2.5 µm^2^ area (128 × 64 pixels), approximately in the same surface region, which could be maintained by in situ annealing. However, the same surface area could not be repeatedly imaged after successive annealing, due to tolerances during probe removal and remount, and to thermal drift during the heating and cooling of the sample. All chitosan samples were fixed to steel disk AFM holders using a solvent‐free two‐component epoxy conductive glue (Epotek, H20E, Epoxy Technology Inc., Billerica, MA, USA) to avoid any degassing even during sample scans at moderate temperatures. Average *d*_33_ and surface roughness values and their uncertainties were determined by statistics on several images taken in different areas of the films.

The total scan time was around 30 min to acquire PFM images at a scan rate of 0.02 lines/s, which could not be increased, to ensure correct CE‐FM‐PFM performance. Regarding the electrostatic background, it should be remarked that its amount can be influenced by the contact potential difference (*V*_CPD_) between the sample surface and the probe tip. The *V*_CPD_ is subject to changes due to probe contamination and to static surface charge or local surface potential variations. Probe contamination usually shows up as a sudden change in the PFM signal during scanning. The amount of static charge and the surface potential may be influenced by different types of sample processing (e.g., chemical, thermal). However, as it is evident from all PFM maps of Figures 6 and 7, during PFM scans the signals remained fairly constant, even up to several hours, to witness the strong immunity of CE‐FM‐PFM to electrostatic effects. Therefore, the same *V*_dc_ value of 0 V was used in all measurements.

Furthermore, another possible contribution to the recorded electromechanical yield could be present, namely, electrochemical, or Vegard, strain [35]. The applied electric field could drive ionic species contained in the sample to migrate toward the surface and correspondingly create an internal electric polarization that could induce an electrostrictive effect, with a similar mechanism to that in ferroelectric materials due to their remnant polarization [35]. It should be expected that Vegard effect decreases after heating of the sample, due to the evaporation of the solvent, as the presence of solvent increases conductivity of ionic species, as also confirmed by conductivity measurement performed by BDS on the same films [33]. Since we do not observe a monotonically decreasing PFM signal with increasing annealing temperature, we could exclude the influence of Vegard strain, at least as the dominant phenomenon. Furthermore, typical conductivity of our samples remains low, of the order of 10^−11^ S/cm in the temperature regime of this study [30]. compared with those of ionic electrolytes or even semiconductors [35] where Vegard strain is dominant.

The dependence of the PFM signal from the amplitude of the oscillating electric potential is documented in Figure S5, showing a fairly linear behaviour, confirming the negligible influence of Vegard strain.

In addition, a further confirmation of the dominance of piezoelectric effect over Vegard strain was obtained by recording piezoloops obtained by sweeping the DC bias *V*_dc_ during a PFM measurement at fixed positions. Linear behavior, with about no hysteresis, was recorded (see Figure S6). This is consistent with the effect of piezoelectricity, but not with Vegard strain.

### 4.7 XRD

The XRD data were acquired by an X’pert Pro MRD Diffractometer (Malvern‐Panalytical, London, UK). The diffractometer has a horizontal *ω*‐2*θ* goniometer (320 mm radius) in a four‐circle geometry, and it works with a ceramic X‐ray tube with Kα anode (*λ* = 1.540 Å). This diffractometer is equipped with a parabolic mirror with a standard divergence slit (1/4°). The detector is a *PIXcel*, a fast X‐ray detector based on Medipix2 technology with a 256 × 256 pixel array. Data were acquired in a *2θ* range from 5° to 35°, with a step size of 0.03° and a counting time/time per step of 245 s. The XRD peak fits were performed using a double Lorentzian function, by the Origin software. The fit amplitudes were used to calculate the peaks intensity ratio. For the L0 sample, the peak amplitude at 10° was set to 0.

## Supporting Information

Additional supporting information can be found online in the Supporting Information section. **Supporting** **Fig. S1**: Photographs showing the main steps in the fabrication of chitosan films. a) The solution is poured into a Petri dish; b) After drying, the film is immersed in a NaOH solution; c) After the neutralization process, the film is placed to dry on a Petri dish put upside‐down. **Supporting Fig. S2**: Stress‐strain curve for a) A0, b) A60, c) F0, d) F60, e) L0, d) L60 samples. **Supporting Fig. S3**: Chitosan films made with acetic, formic, and lactic acid, without NaOH treatment (A0, F0, L0) and after 60 minutes of NaOH treatment (A60, F60, L60), after being subjected to annealing at different temperatures: 100°C, 140°C, 200°C, 250°C. The annealing temperatures (in °C) are indicated at the top of the figure. **Supporting Fig. S4**: XRD spectra of chitosan films made using a,b) acetic, c,d) formic, and e,f) lactic acid annealed at different temperatures. Panels a), c), and e) show the spectra of samples without NaOH treatment, while panels b), d), and f) correspond to NaOH‐treated samples. The annealing temperature for each spectrum is indicated in the label. **Supporting Fig. S5**: Dependence of PFM signal on Vdc (piezoloop) on A60_140 sample, with Vac = 1VRMS. **Supporting Fig. S6**: Dependence of PFM signal on Vac for the A60_140 sample. **Supporting Table S1**: Intensity ratio between the NH_2_ and NH_3_^+^ vibrational modes for each sample from FTIR‐ATR spectra. The corresponding value for the Chitosan powder is 1.5. **Supporting Table S2**: Results of TGA: temperature ranges [°C], the peak of degradation [°C], and associated weight losses [%] of non‐NaOH‐treated as well as 60 min ‐NaOH‐treated samples. **Supporting Table S3**: Degree of crystallinity *C*_d_ (not normalized), *d*_33_ coefficient, and intensity ratio between the XRD peaks at 10° and 20° for each sample.

## Author Contributions

**Sofia Papa**: investigation, visualization, writing – original draft, data curation, formal analysis, methodology, writing – review and editing. **Margherita Montorsi**: conceptualization, investigation, visualization, data curation, formal analysis, methodology, validation, writing – review and editing. **Leonardo Arrighetti**: investigation, data curation, formal analysis, writing – review and editing. **Cristian Rodriguez‐Tinoco**: investigation, data curation, formal analysis, writing – review and editing. **Simone Capaccioli**: validation, writing – review and editing. **Laura M. Ferrari**: supervision, funding acquisition, validation, writing – review and editing. **Francesco Greco**: funding acquisition, project administration, supervision, validation, writing – review and editing. **Massimiliano Labardi**: conceptualization, project administration, investigation, supervision, data curation, formal analysis, resources, methodology, validation, writing – review and editing.

## Funding

F.G. acknowledges the support of the BRIEF ‘Biorobotics Research and Innovation Engineering Facilities’ project (Project identification code IR0000036) funded under the National Recovery and Resilience Plan (NRRP), Mission 4 Component 2 Investment 3.1 of the Italian Ministry of University and Research funded by the European Union – NextGenerationEU. F.G. and L.M.F. acknowledge funding by European Union‐Next Generation EU via the Italian Ministry of University and Research (MUR), PNRR‐ Investment 1.5 Ecosystems of Innovation, Project Tuscany Health Ecosystem (THE), Spoke 3 ‘Advanced technologies, methods, materials and health analytics’ CUP: I53C22000780001.

## Conflicts of Interest

The authors declare no conflicts of interest.

## Supporting information

Supplementary Material

## Data Availability

The data that support the findings of this study are available from the corresponding author upon reasonable request.
