# Supplementary material for: A Comprehensive Study of Piezoelectricity in Chitosan Films
Source: Small Sci. 2026 Mar 23;6(3):e202500593. doi: 10.1002/smsc.202500593 (PMC13098043; doi:10.1002/smsc.202500593)
Supplement: Supplementary file 1 — Supplementary Material [file SMSC-6-e202500593-s001.pdf]

Supporting Information

A Comprehensive Study of Piezoelectricity in Chitosan Films

Sofia Papa[1,+], Margherita Montorsi[2,3,+], Leonardo Arrighetti[2,4], Cristian Rodriguez-Tinoco[5], Simone Capaccioli[2,6], Laura M. Ferrari[1*], Francesco Greco[1,7*,], Massimiliano Labardi[2*]

1. *The Biorobotics Institute and Dept. of Excellence in Robotics & AI, Scuola Superiore Sant'Anna, Viale R. Piaggio 34, 56025 Pontedera, Italy*
2. *Consiglio Nazionale delle Ricerche, Istituto per i Processi Chimico-Fisici (CNR-IPCF), Sede Secondaria di Pisa, Via Moruzzi 1, 56124 Pisa, Italy*
3. *College of Physics and Optoelectronics Engineering, Shenzhen University, Shenzhen 518060, People's Republic of China*
4. *Dipartimento di Chimica e Chimica Industriale, Università di Pisa, Via G. Moruzzi 13, 56124, Pisa, Italy*
5. *Departamento de Física. Facultad de Ciencias, Universitat Autònoma de Barcelona, and Catalan Institute of Nanoscience and Nanotechnology (ICN2), CSIC and BIST, Campus UAB, Bellaterra 08193, Spain*
6. *Physics Department, University of Pisa, Largo Pontecorvo 3, 56127 Pisa, Italy*
7. *Interdisciplinary Center on Sustainability and Climate, Scuola Superiore Sant'Anna, Piazza Martiri della Libertà 33, 56127 Pisa, Italy*

+ These Authors contributed equally to this work.
** Corresponding Authors. Email: laura.mferrari@santannapisa.it, francesco.greco@santannapisa.it, labardi@df.unipi.it*

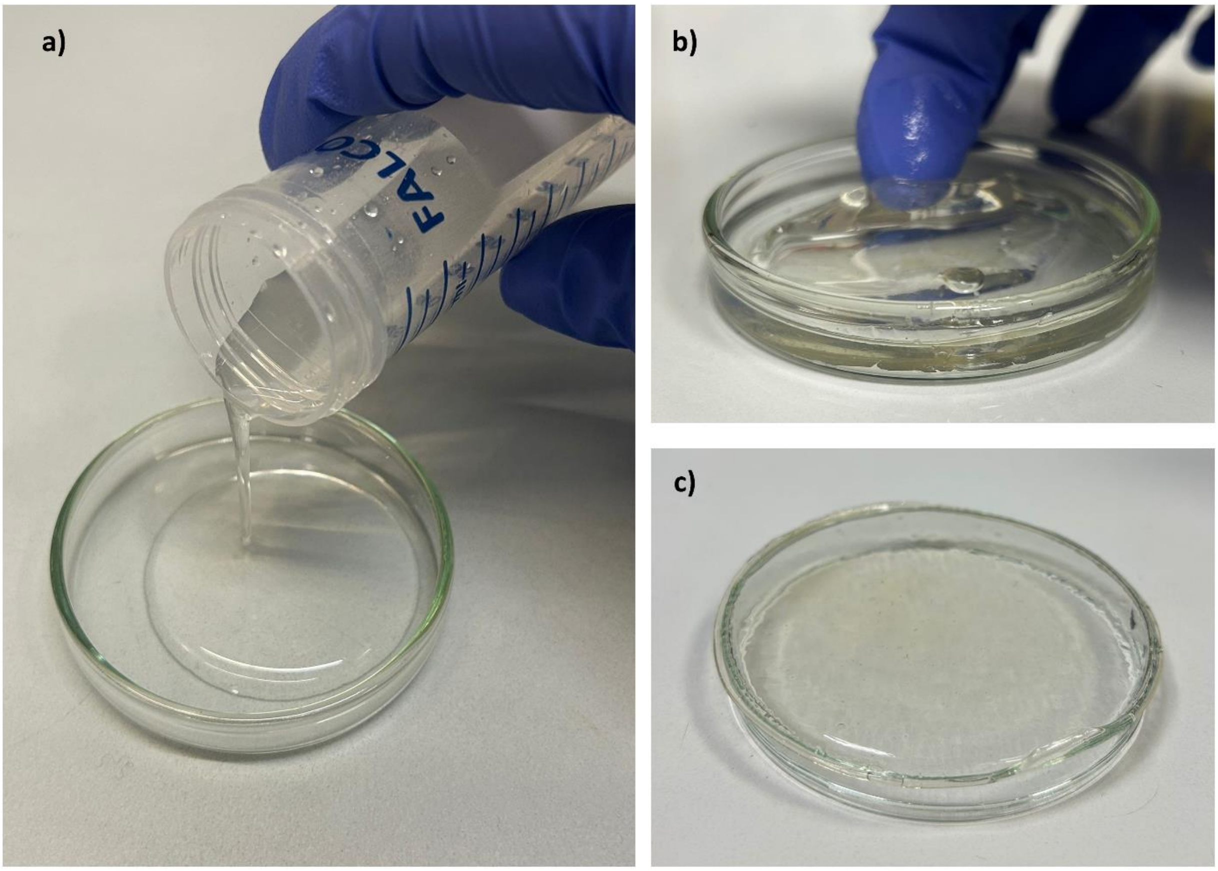

Figure S.1: Photographs showing the main steps in the fabrication of chitosan films. a) The solution is poured into a Petri dish; b) After drying, the film is immersed in a NaOH solution; c) After the neutralization process, the film is placed to dry on a Petri dish put upside-down.

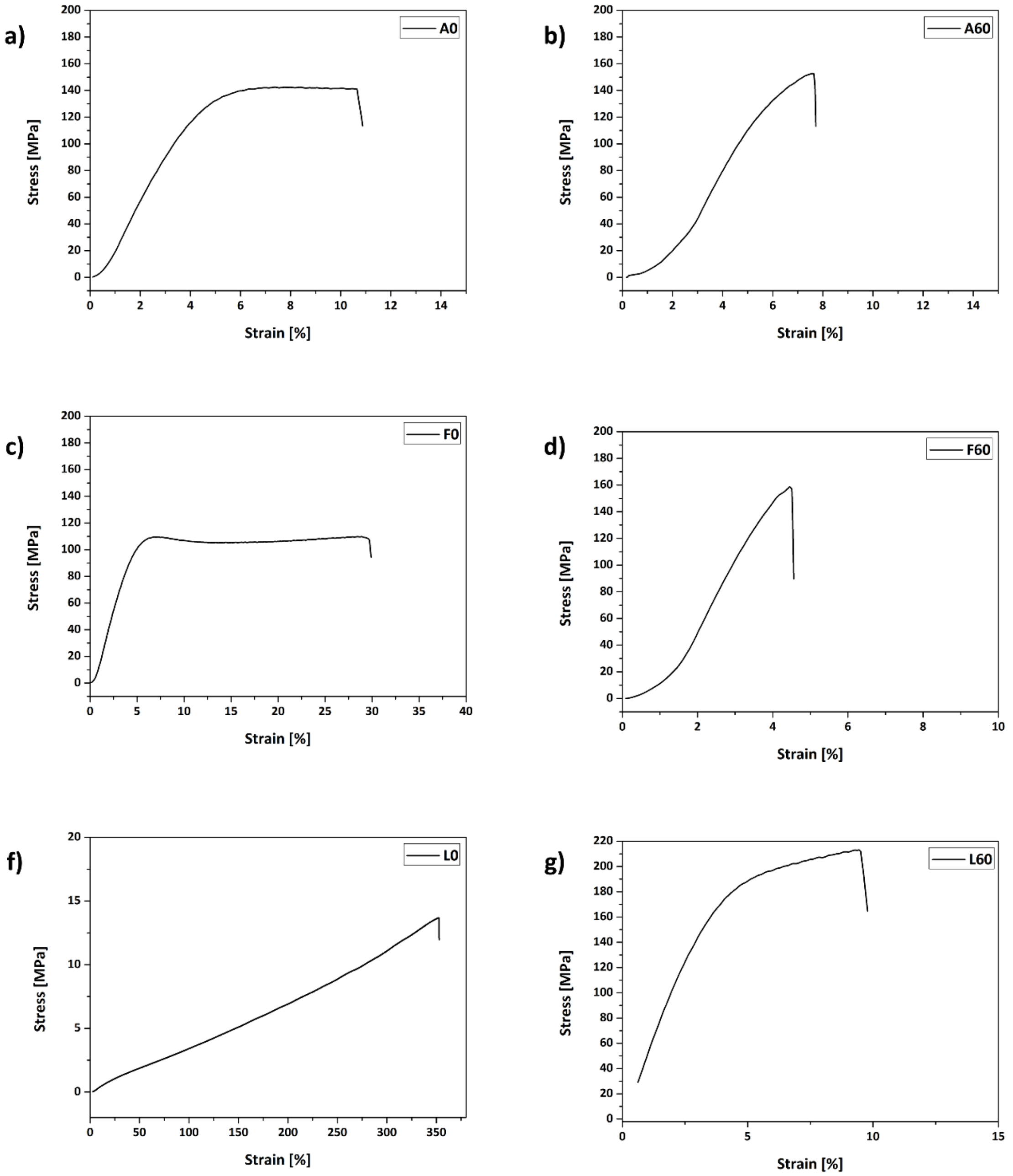

Figure S.2: Stress-strain curve for a) A0, b) A60, c) F0, d) F60, e) L0, d) L60 samples.

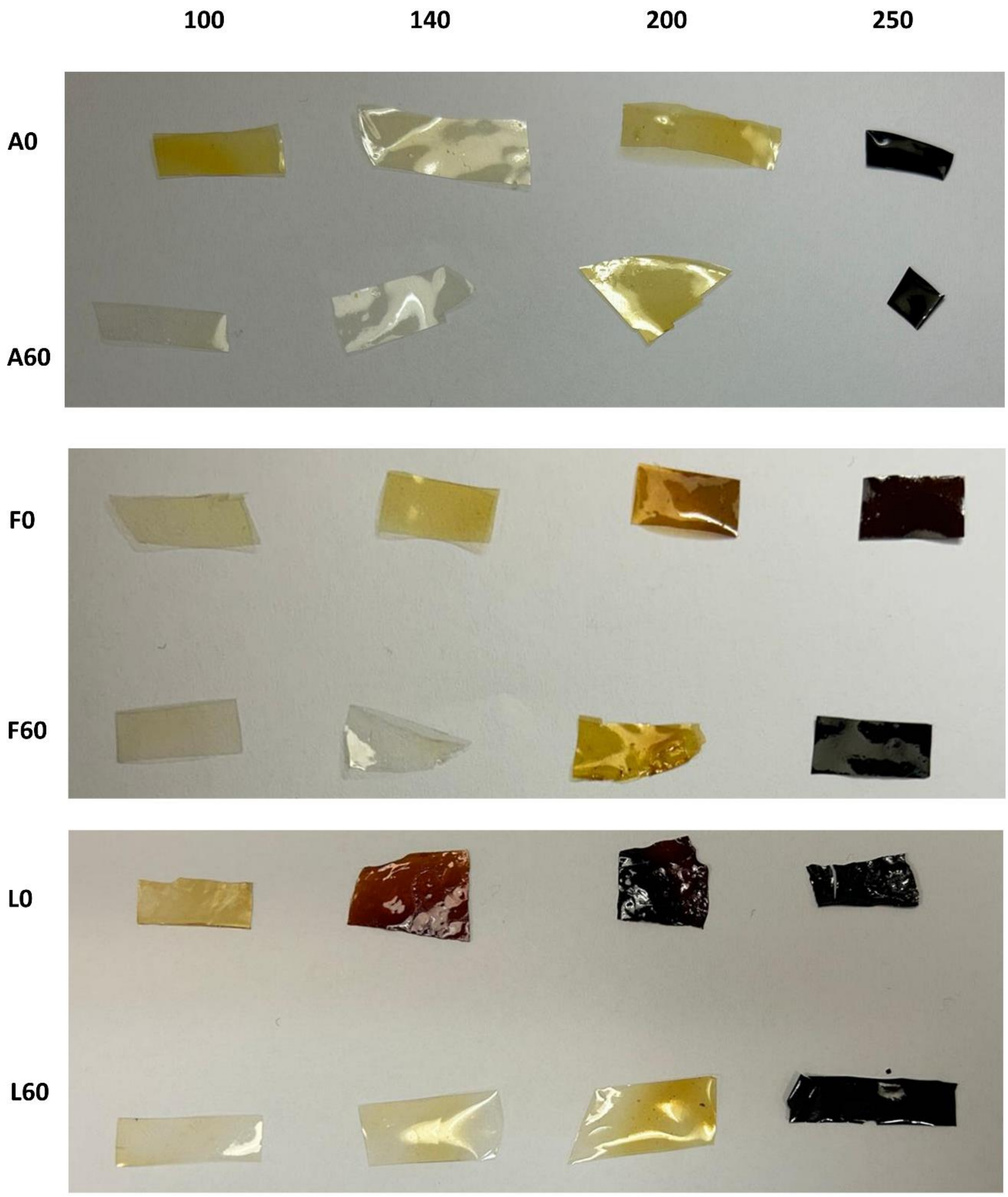

Figure S.3: Chitosan films made with acetic, formic, and lactic acid, without NaOH treatment (A0, F0, L0) and after 60 minutes of NaOH treatment (A60, F60, L60), after being subjected to annealing at different temperatures:100°C, 140°C, 200°C, 250°C. The annealing temperatures (in °C) are indicated at the top of the figure.

Table S.1: Intensity ratio between the NH_2 and NH_3^+ vibrational modes for each sample from FTIR-ATR spectra. The corresponding value for the Chitosan powder is 1.5.

Sample	NH_2/NH_3^+	Sample	NH_2/NH_3^+	Sample	NH_2/NH_3^+
**A0_100**	0.4	**F0_100**	0.5	**L0_100**	0.9
**A0_140**	0.7	**F0_140**	0.5	**L0_140**	0.7
**A0_250**	2.3	**F0_250**	1.7	**L0_250**	1.7
**A60_100**	1.3	**F60_100**	1.4	**L60_100**	1.2
**A60_140**	1.2	**F60_140**	1.5	**L60_140**	1.3
**A60_250**	1.9	**F60_250**	1.9	**L60_250**	1.9

Table S.2. Results of TGA: temperature ranges [°C], the peak of degradation [°C], and associated weight losses [%] of non-NaOH-treated as well as 60 min -NaOH-treated samples.

	Sample	Temp. range [°C]	Peak [°C]	Weight loss [%]	Sample	Temp. range [°C]	Peak [°C]	Weight loss [%]
**1st Stage**	**powder**	25-220	70	9	___	___	___	___
	**F0**	25-220	160	12	**F60**	25-220	77	10
	**A0**	25-220	165	12	**A60**	25-220	65	10
	**L0**	25-220	182	40	**L60**	25-220	70	11
**2nd Stage**	**powder**	220-400	298	49	___	___	___	___
	**F0**	220-400	302	49	**F60**	220-400	293	43
	**A0**	220-400	294	45	**A60**	220-400	292	44
	**L0**	220-400	291	38	**L60**	220-400	290	43
**3rd Stage**	**powder**	400-650	513	36	___	___	___	___
	**F0**	400-650	530	39	**F60**	400-650	578	37
	**A0**	400-650	561	39	**A60**	400-650	570	37
	**L0**	400-650	578	22	**L60**	400-650	562	29

Table S.3: Degree of crystallinity C_d (not normalized), d_{33} coefficient, and intensity ratio between the XRD peaks at 10° and 20° for each sample.

Sample	C_d	d_{33} [pm V^{-1}]	I_{10}/I_{20}
A0_100	1.77	4.5±0.5	0.76
A0_140	1.91	5.8±1.7	0.47
A0_200	2.692	5.9±0.1	0.35
A0_250	4.52	3.7±2.7	0.13
A60_100	2.45	14.8±5.3	0.98
A60_140	2.39	11.7±5.4	1.53
A60_200	2.18	18.0±4.3	1.16
A60_260	1.96	9.5±2.9	1.49
F0_100	2.58	4.3±2.2	0.41
F0_140	3.78	8.1±3.8	0.18
F0_200	3.44	4.8±2.5	0.29
F0_250	3.76	4.6±0.8	0.31
F60_100	2.42	9.6±3.2	1.54
F60_140	2.31	17.3±10.4	1.34
F60_200	1.77	7.0±2.2	1.13
F60_250	2.00	6.6±4.6	1.03
L0_100	2.22	1.9±0.6	0.05
L0_140	2.58	3.0±0.3	0.04
L0_200	3.32	5.0±0.1	0.03
L0_250	1.74	9.3±2.8	0.07
L60_100	2.66	8.7±1.0	2.10
L60_140	2.93	27.3±3.7	0.80
L60_200	2.66	4.9±1.4	1.10
L60_250	1.88	7.8±5.2	1.68

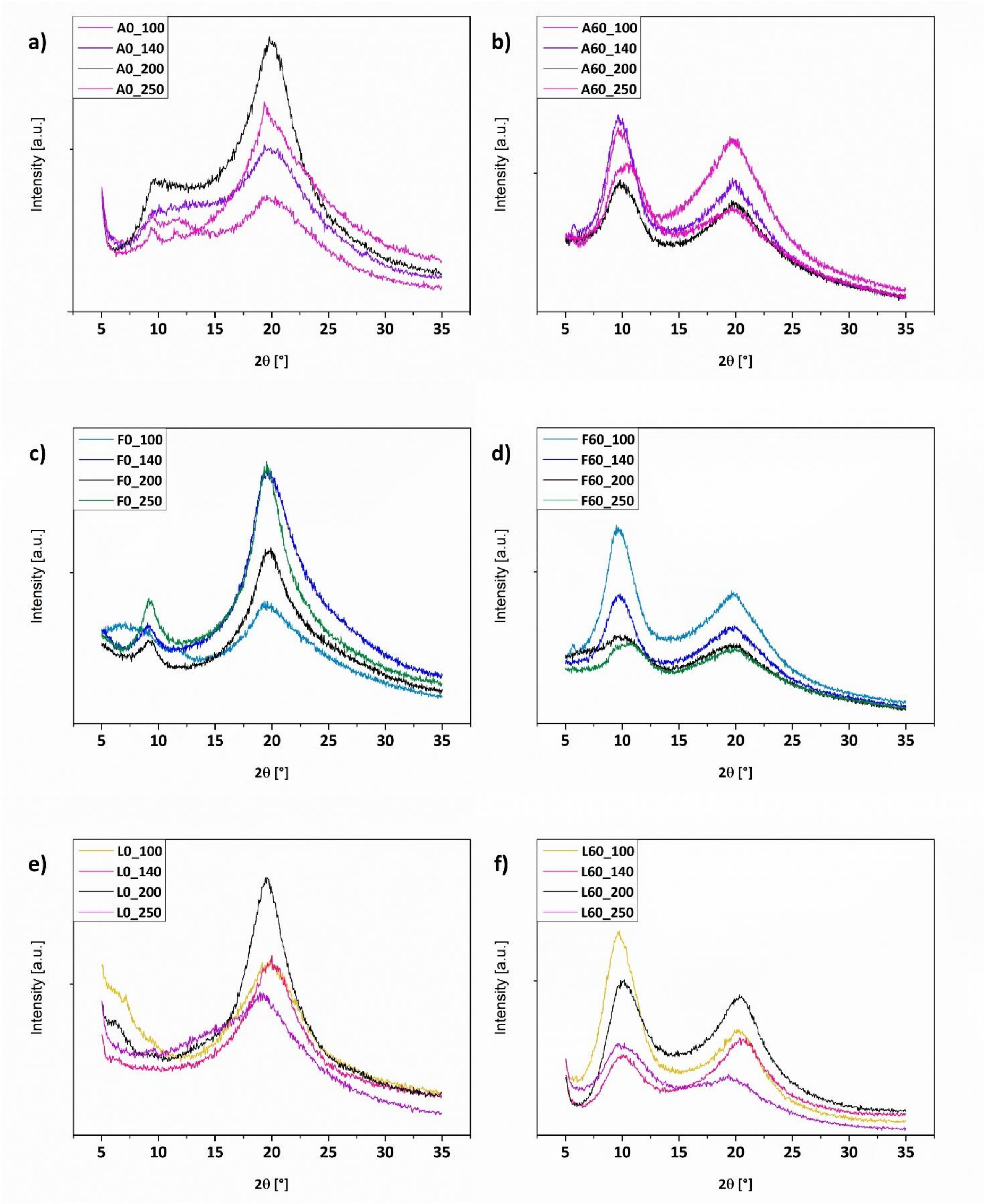

Figure S.4: XRD spectra of chitosan films made using a,b) acetic, c,d) formic, and e,f) lactic acid annealed at different temperatures. Panels a), c), and e) show the spectra of samples without NaOH treatment, while panels b), d), and f) correspond to NaOH-treated samples. The annealing temperature for each spectrum is indicated in the label.

Experimental methodology for local piezoresponse measurements

To measure electromechanical effects by PFM, an electric potential is applied to a conductive AFM probe, generating an electric field inside the sample, and for piezoelectric materials, this produces a surface deformation, that can be measured as a force acting on the AFM probe itself. In addition, an electrostatic force is also present, due to the Coulombic interaction of surface charges with the biased probe, even for non-piezoelectric samples. Also, electromechanical effects different from piezoelectricity could play a role, like for instance Vegard strain. [1] Careful control and analysis of measurements is necessary to discriminate the effects of these different contributions, and derive the correct piezoelectric yield of the surface.

Constant-excitation frequency-modulation (CE-FM) PFM [2] was developed to achieve a fairly good degree of discrimination between actual piezoelectricity and electrostatic artifacts. The method is summarized in the following. The main source of measurement artifacts in PFM methods is the cantilever bending due to the electrostatic force arising between probe and sample, occurring when applying the necessary electric potential in order to stimulate piezoelectricity. Even if the sample is not piezoelectric, such bending mimics indeed a surface displacement, because the probe/surface distance is modified by the displacement of the probe instead of that of the surface. If the sample is highly stiff, as for perovskite ceramics, and the probe is in static contact with the surface, electrostatic bending is contrasted by the repulsive atomic force, and therefore, as it is well known, electrostatic artifacts do not affect much contact-mode PFM performed on stiff materials. On the other hand, for softer materials, like polymers and organic samples, the cantilever bending can be remarkable, since it is allowed by indentation of the soft material, especially if the spring constant is low as for contact-mode cantilevers.

If the cantilever is stiff, the electrostatic force produces much less bending, and correspondingly, less indentation. Therefore, use of stiff cantilevers is an effective method to reduce electrostatic artifacts with soft materials.

Dynamic modes of the AFM, like the so-called tapping mode, are widespread methods to reduce surface damage due to excessive contact force, as well as wear phenomena and jump-in-contact instabilities and reduce thermal and measurement noise. All this is possible because of the employment of stiff cantilevers and exploiting the resonant effect of the cantilever, used as a simple harmonic oscillator. In the tapping mode, the probe swings normally to the surface, and enters the repulsive regime only for a portion of the total oscillation period. This reduces the average probe/sample force, albeit still retaining the high resolution allowed by the repulsive AFM interaction. Therefore, damage to soft materials is reduced, and nanoparticles or other

nanostructures, that might be poorly adhered to the substrate, can be studied without being dragged around like occurring in the contact mode.

Piezoelectric deformation of the surface in the direction of the symmetry axis of the probe (z) is determined by the d_{33} piezoelectric coefficient times the z component of the electric field inside the material. When such surface deformation occurs, the probe/sample distance is changed, and therefore the atomic forces change as well, thereby influencing the oscillatory motion of the probe. It can be shown [3] that two different effects occur, namely, a shift of the oscillator resonant frequency, due mainly to the repulsive atomic force, and a change of the oscillator damping, due mainly to energy dissipation at the molecular layers adsorbed on the surface.

In tapping mode, the oscillation frequency is kept fixed by the system. For this reason, both resonance shift and damping phenomena cause a change of the oscillation amplitude, and therefore, their discrimination is difficult. That is the reason why tapping mode has rarely been exploited in PFM.

In frequency-modulation (FM) AFM [4] the oscillation frequency is changed by the system in real time, in such a way that the oscillator is kept at resonance at all times. Oscillation frequency and amplitude are measured separately. Conservative forces, like the repulsive atomic force as well as the electrostatic force, are only able to produce a resonance shift, but are unable to change the amplitude of the oscillation, because they cannot dissipate energy. In contrast, surface dissipation phenomena are only able to change the oscillation amplitude, but do not sensibly affect the resonant frequency. In conclusion, by using FM-PFM, the surface displacement caused by piezoelectricity can be discriminated from the electrostatic contribution, since the former can produce both a frequency shift (because of the atomic repulsive force change) and an amplitude damping (because of the surface dissipation change), while the latter can only produce a frequency shift. Therefore, the measurement of an amplitude change in FM-PFM can only be due to a surface displacement, and therefore, to the piezoelectric effect.

The adoption of the constant-excitation (CE)-FM mode is dictated by the necessity to allow the change of oscillation amplitude by virtue of the surface displacement. An alternative mode is named constant-amplitude (CA)-FM, that presents advantages in terms of reaction speed of the measurement, and is mainly used in ultra-high vacuum AFM experiments, while it provides no advantage compared to CE-FM for use in ambient.

The CE-FM-PFM method can be easily implemented on an atomic force microscope operating in CE-FM mode. Topography feedback should be stabilized at constant amplitude (A), similarly than in tapping mode. Amplitude setpoint should be kept in a range between 50% and 75% of

free amplitude A_0. The probe should be biased by a potential $V(t) = V_{dc} + V_{ac}\cos(\Omega t)$, with $f = \Omega/2\pi$ of around 100-200 Hz for the best operation of the method [2]. Indeed, by adopting a low gain value for the topographic feedback adjustment, surface displacement by piezoeffect cannot be compensated by the feedback action at such frequency values, that result to be too high to be followed [2].

Either the Δf or ΔA outputs of the CE-FM-AFM are sent to a lock-in amplifier, referenced to the frequency f. Outputs of the lock-ins correspond to the instantaneous oscillation resonant frequency and amplitude, due to the action of the AC potential. Specifically, in CE-FM-PFM the amplitude change is identified with the surface displacement due to the piezoelectric effect. To calibrate the amplitude channel and therefore the piezoelectric displacement, the feedback loop amplitude setpoint is changed by a known fraction, and the corresponding change of topographic height (Δz) is recorded. Since z is calibrated (in nm), also A can be calibrated.

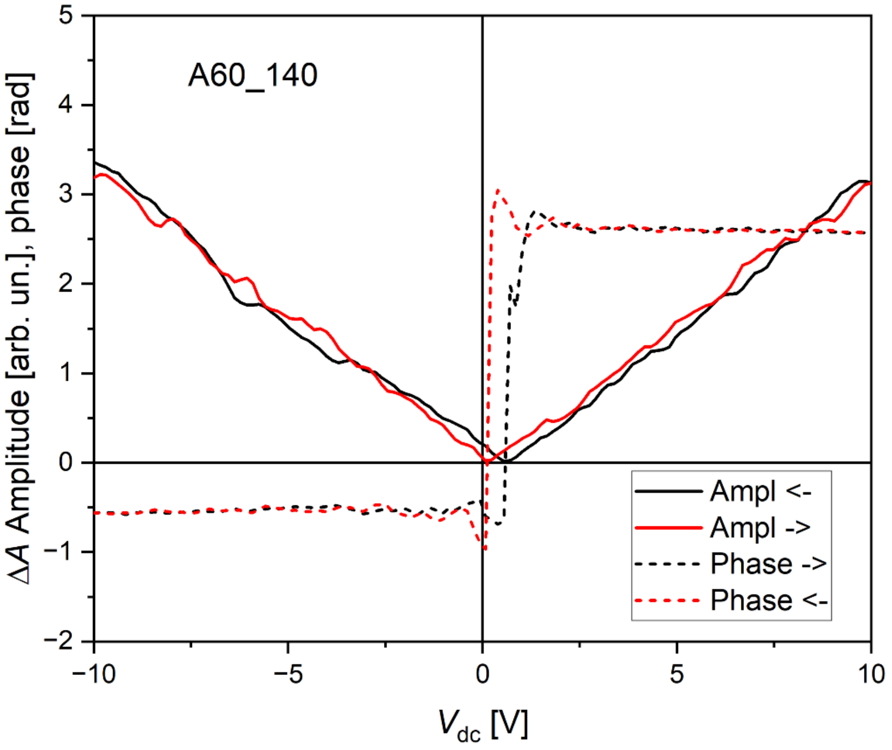

Figure S.5: Dependence of PFM signal on V_{dc} (piezoloop) on A60_140 sample, with $V_{ac} = 1V_{RMS}$.

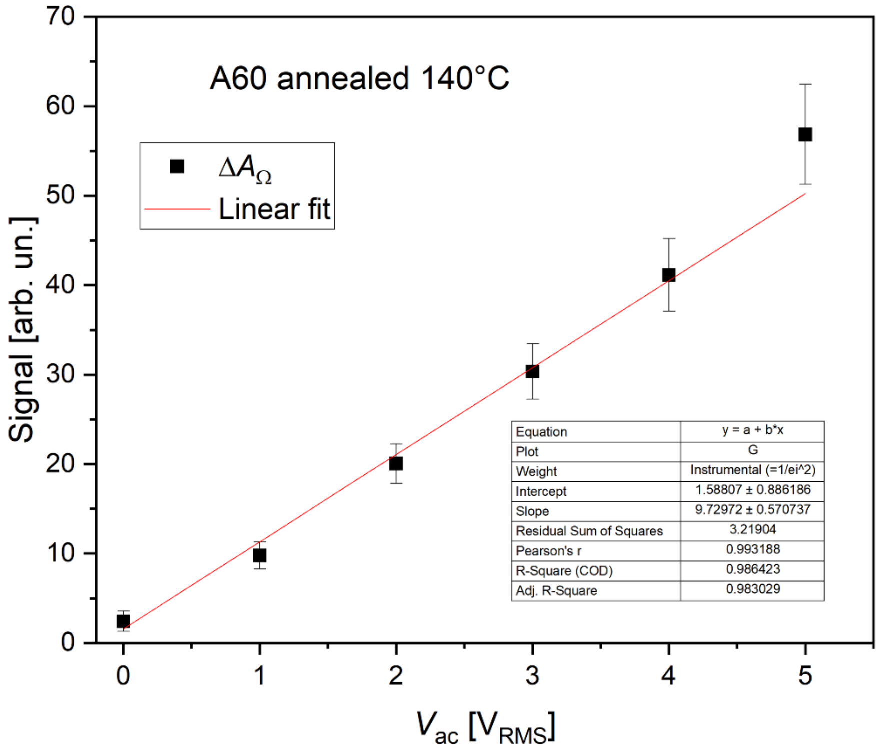

Figure S.6: Dependence of PFM signal on V_{ac} for the A60_140 sample.

References.

[1] Vasudevan, R. K., et al., Ferroelectric or non-ferroelectric: Why so many materials exhibit "ferroelectricity" on the nanoscale. Applied Physics Reviews 4.2 (2017)

[2] Labardi, M., et al., Piezoelectric displacement mapping of compliant surfaces by constant-excitation frequency-modulation piezoresponse force microscopy. Nanotechnology 31.7 (2019): 075707

[3] V.V. Protasenko, M. Labardi, and A.C. Gallagher, Conservative and dissipative forces measured by self-oscillator atomic force microscopy at constant-drive amplitude. Physical Review B 70 (2004): 245414.

[4] T.R. Albrecht, P. Grütter, D. Horne, and D. Rugar, Frequency modulation detection using high-Q cantilevers for enhanced force microscope sensitivity. Journal of Applied Physics 69 (1991): 668–673.
